# Safety and Immunogenicity of a Malaria Vaccine, *Plasmodium falciparum* AMA-1/MSP-1 Chimeric Protein Formulated in Montanide ISA 720 in Healthy Adults

**DOI:** 10.1371/journal.pone.0001952

**Published:** 2008-04-09

**Authors:** Jinhong Hu, Zhihui Chen, Jun Gu, Mobin Wan, Qian Shen, Marie-Paule Kieny, Jia He, Zhen Li, Qingfeng Zhang, Zarifah Hussain Reed, Yongmei Zhu, Wenjie Li, Yang Cao, Li Qu, Zhifang Cao, Qiang Wang, Haitao Liu, Xuegong Pan, Xiudong Huang, Dongmei Zhang, Xiangyang Xue, Weiqing Pan

**Affiliations:** 1 Changhai Hospital, Second Military Medical University, Shanghai, China; 2 Department of Pathogen Biology and State Key Laboratory of Medical Immunology, Second Military Medical University, Shanghai, China; 3 Department of Statistics, Second Military Medical University, Shanghai, China; 4 Shanghai Wanxing Biopharmaceuticals Co., Ltd., Shanghai, China; 5 Initiative for Vaccine Research, World Health Organization, Geneva, Switzerland; Queensland Institute of Medical Research, Australia

## Abstract

**Background:**

The P. falciparum chimeric protein 2.9 (PfCP-2.9) consisting of the sequences of MSP1-19 and AMA-1 (III) is a malaria vaccine candidate that was found to induce inhibitory antibodies in rabbits and monkeys. This was a phase I randomized, single-blind, placebo-controlled, dose-escalation study to evaluate the safety and immunogenicity of the PfCP-2.9 formulated with a novel adjuvant Montanide ISA720. Fifty-two subjects were randomly assigned to 4 dose groups of 10 participants, each receiving the test vaccine of 20, 50, 100, or 200 µg respectively, and 1 placebo group of 12 participants receiving the adjuvant only.

**Methods and Findings:**

The vaccine formulation was shown to be safe and well-tolerated, and none of the participants withdrew. The total incidence of local adverse events (AEs) was 75%, distributed among 58% of the placebo group and 80% of those vaccinated. Among the vaccinated, 65% had events that were mild and 15% experienced moderate AEs. Almost all systemic adverse reactions observed in this study were graded as mild and required no therapy. The participants receiving the test vaccine developed detectable antibody responses which were boosted by the repeated vaccinations. Sixty percent of the vaccinated participants had high ELISA titers (>1∶10,000) of antigen-specific antibodies which could also recognize native parasite proteins in an immunofluorescence assay (IFA).

**Conclusion:**

This study is the first clinical trial for this candidate and builds on previous investigations supporting PfCP-2.9/ISA720 as a promising blood-stage malaria vaccine. Results demonstrate safety, tolerability (particularly at the lower doses tested) and immunogenicity of the formulation. Further clinical development is ongoing to explore optimizing the dose and schedule of the formulation to decrease reactogenicity without compromising immunogenicity.

**Trial Registration:**

Chinese State Food and Drug Administration (SFDA) 2002SL0046; Controlled-Trials.com ISRCTN66850051 [66850051]

## Introduction

The emergence of drug-resistant parasites and insecticide-resistant Anopheles mosquitoes have contributed to the persistence of malaria in the world [1], [2], [3]. New methods to control the disease are needed, and vaccination holds the promise of controlling and perhaps eventually eradicating malaria.

The asexual blood stage is responsible for malaria disease. Naturally acquired immunity develops against disease in malaria-endemic regions, where parasite density is observed to decrease with age and the clinical manifestations of malaria are generally much milder in adults than in children under 5 years of age [4], [5]. Moreover, immunoglobulin fractions from adults in these high-transmission areas have been found to passively reduce parasitemia in infected children [6], [7].Thus an effective vaccine targeting this stage and inducing immune responses similar to that obtained by natural infection could reduce morbidity and mortality of the disease.

Several antigens thought to be targets of protective blood-stage immune responses have been identified [8], [9], [10]. Among them, the ∼200 kDa Merozoite Surface Protein 1 (MSP1) and the Apical Membrane Antigen 1 (AMA-1) of *Plasmodium falciparum* are two leading asexual blood-stage vaccine candidates [10]. Located on the merozoite surface, they are proposed to play a role in the process of parasitic invasion [11], [12]. A portion of MSP1 recognized by protective antibodies was mapped to the 19 kDa carboxy-terminal region (MSP1-19) which contains two epidermal growth factor (EGF)-like domains [13]. Immunization with MSP1-19 of P. falciparum in monkeys and P. yoelii in mice induced protection against homologous parasite challenge [14], [15]. Data from these animal studies were supported by in vitro studies showing that MSP1-19 specific antibodies inhibited merozoite invasion [16].

AMA-1 is an integral membrane protein of 83 kDa. Studies in rodent and monkey models demonstrated that immunization with native or recombinant AMA-1 can provide protection against homologous parasite challenge [17]. A three-domain sub-structure of the AMA1 ectodomain was recently suggested [18]. The most C-terminal of the disulphide-bonded domains in AMA-1 (domain III) containing a “cysteine knot-like” structure may be carried on the surface of the invading merozoite along with MSP1-19. Studies with P. chabaudi in a mouse model have shown that antibody-mediated protection can be induced by immunizing with the AMA1 ectodomain [19]. The structure of AMA-1 (III) was recently demonstrated to be the target of inhibitory antibodies isolated from humans in malaria-endemic regions [20]. A recent study showed that AMA-1(III) bound to the erythrocyte membrane protein, Kx, and that the subsequent rate of invasion of Kx null erythrocytes was reduced, suggesting that AMA-1 (III) plays an important role in merozoite invasion of human erythrocytes [21].

Both MSP1-19 and AMA-1 (III) can be a target of inhibitory antibodies, but the small size of the antigens may limit the ability of each alone to adequately induce the high titer of antibodies that may be required to be effective in vivo. Polyvalent subunit malaria vaccines containing multiple protective domains or epitopes from different antigens may be necessary for enhanced immunogenicity and protective efficacy and may be needed to address the issue of parasite variation. Chimeric protein vaccine constructs may be an approach towards this and therefore, we have constructed a P. falciparum chimeric protein 2.9 (PfCP-2.9) by combining MSP1-19 and AMA-1 (III) sequences [22]. The purified PfCP-2.9 protein, produced by Pichia pastoris in secreted form with a yield of 2,600 mg/L, was highly immunogenic in rabbits as well as in rhesus monkeys (Macaca mulatta). It induced both anti-MSP1-19 and anti-AMA-1 (III) antibodies at 11- and 18-fold higher titers, respectively, compared to individual components. Anti-PfCP-2.9 sera from rabbits and rhesus monkeys at a 6 to 7-fold dilution nearly completely inhibited in vitro growth of the P. falciparum FCC1/HN and 3D7 strains [22]. The inhibition was dependent on the presence of antibodies to the chimeric protein and their disulfide bond-dependent conformations. Moreover, the activity was mediated by a combination of growth-inhibitory antibodies generated by the individual MSP1-19 and AMA-1 (III) components of PfCP-2.9.

In this study, we report the first human phase I trial of recombinant PfCP-2.9 formulated with the novel adjuvant Montanide ISA720 [23], [24].

## Methods

### Study Setting

The Phase I trial was a randomized, single blinded, placebo-controlled, dose-escalation study conducted at theChinese National Medicament Clinical Study Base, Changhai Hospital in Shanghai, China. The protocol for this trial and supporting CONSORT checklist are available as supporting information; see Checklist S1 and Protocol S1.

### Participants

Seventy-two healthy malaria-naïve adults were screened after have given written informed consent. Exclusion criteria included a history of malaria or positive markers for antibodies to malaria parasite by IFA; history of traveling to or residing in a malaria endemic region within the last two years; history of allergic reactions or convulsions following vaccination; acute illness within four weeks prior to the trial; involvement in any other drug or vaccine trial within four weeks prior to enrollment; presence of any chronic disease or HBV (HBsAg) and HCV infection; pregnancy; abnormal hematology and clinical chemistry and elevated levels of anti-nuclear antibodies(>160). 62 volunteers were found eligible and enrolled. They were randomly allocated as 52 participants in 5 groups and 10 standbys. 4 groups (10 participants each group) received the vaccine formulation of either 20 µg, 50 µg, 100 µg or 200 µg while the fifth (12 participants) received placebo.

### Intervention

The PfCP-2.9 chimeric protein was constructed and produced in *P. pichia* as described before[22]. Briefly, the redesigned synthetic gene encoding the chimeric protein was expressed in the yeast system in a secreted form at an extremely high yield of 2.6 g/L in a 15-liter fermentation run. The recombinant protein resembled closely its native protein as all six conformational monoclonal antibodies interacted with the protein. To prepare for this study, three batches of the protein (lots No. 20010501∼03) were produced at the 30-L scale at the Pilot Bioproduction Facility of Shanghai Wanxing Bio-pharmaceutical Co. Ltd. The conditions for the fermentation of the construct were optimized to achieve high level expression. The protein was purified through hydrophobic interaction, ion-exchange and gel filtration chromatography. The purified protein was a single band and more than 99% pure as measured by SDS-PAGE and HPLC. It contained <2.5 endotoxin units per dose and <0.1% host protein. In addition, N-terminal sequences of the purified protein were determined by Edman degradation from the first residue to position 15 which found the determined sequence to be identical to the designed sequence. The protein reacted with the conformational monoclonal antibody mAb5.2 recognizing MSP1-19 (see Data S1 of this paper).

The purified protein was formulated with the Montanide ISA720 adjuvant by mixing 70% volume of the adjuvant with 30% volume of the antigen solution using a Homogeneizer at 4000 rpm for 4 min at room temperature. The parameters for homogenization of the vaccine emulsion were determined in prior experiments to achieve an acceptable size of approximately 1 µm diameter(the manufacturer of the ISA 720 adjuvant kindly carried out this detection using laser diffraction-based particle size analyzer) although the vaccine emulsion lots used in the clinical trial were not analyzed for droplet size distribution. The quality of the emulsion was controlled by several parameters including droplet test and conductivity. The formulated vaccine was characterized as follows: conductivity (25°C) was about 0.1 µs, viscosity (25°C) about 15 mPas and microscopic appearance was homogeneous dispersion of small and regular droplets (about 1 µm in diameter). After passing sterility tests, the vaccine was packaged into autoclaved 2 mL bottle with 1 mL volume of emulsion. Similarly, the comparator emulsion used for placebo control was prepared by mixing 70% volume of the adjuvant with 30% volume of the PBS solution. To assess physical and biochemical stability, SDS-PAGE gel with Commas-blue and silver staining, Western blot as well as a sandwich ELISA-based method developed by us were utilized on the vaccine emulsion lots used in the pre-clinical study (manuscript in preparation). In addition potency was evaluated with immunogenicity studies in mice (ED_50_). Vaccine emulsions were stored at 4°C and tested at regular intervals. The results showed that the vaccine was stable for at least one year and a half, although the aggregation of the protein was observed during the storage at 4°C. There were no significant changes of the potency and GIA efficacy of the stored emulsion compared with the fresh emulsion. Moreover, the nature of the protein in the emulsion was detected by the sandwich ELISA method which showed that the conformation of the protein in the emulsion after 18 months was not changed. Based on the stability and potency of the pre-clinical vaccine formulation lots, we developed a protocol to produce four lots of 400 µg, 200 µg, 100 µg and 40 µg of the vaccine formulation for use in the clinical study. Physical and biochemical stability and potency of the clinical vaccine lots were evaluated for six months. The results showed that all the clinical lots of the formulation were stable during the period of six months. No degraded band of this protein was detected by SDS-PAGE with Commas-blue staining and the ED_50_ of the vaccine emulsion stored for 0, 3 and 6 months at 4°C were 0.037, 0.032 and 0.033 µg, respectively, which showed no significant changes between the fresh formulation (0 month) and post-storage formulation for 3 and 6 months ( see Data S1 of this paper).

On day 0, three participants in the 20 µg group and one in the placebo group were injected intramuscularly in the left lateral deltoid with 0.5 mL of vaccine or placebo formulation, respectively. Following a review of safety data by the study Data Safety Monitoring Board (DSMB), after a two week interval, the remaining participants from the 20 µg group and placebo were injected. A similar review and 2 week interval preceded before injecting the next group at higher doses.. The participants in rest of the groups were immunized in a similarly staged manner. The vaccinations were repeated on Day 60 in the right lateral deltoid and on Day180 in the left one.

### Clinical Oversight, Ethical and Regulatory Approval

The sponsor, World Health Organization, convened a study team which reviewed the trial protocol and monitored the study conduct on site regularly. Safety data from the study was evaluated by the Data Safety Monitoring Board (DSMB). Ethical approval was obtained by Independent Ethics Committee (IEC) of Shanghai Changhai Hospital and the WHO Ethical Review Committee (ERC) (then known as Secretariat Committee for Research Involving Human Subjects).

The vaccine candidate was approved for clinical trial by the State Food and Drug Administration in China (SFDA).

### Study design and procedures

#### Objectives

The objectives of the study were to evaluate the safety, reactogenicity and immunogenicity of the PfCP-2.9 recombinant vaccine adjuvanted with Montanide ISA 720 (SEPPIC, France) in healthy adults.

#### Outcomes

Safety and Reactogenicity: After each injection participants were observed for 24 h to identify and treat immediate reactions and symptoms. Diary cards were distributed and participants were instructed to record adverse events daily for 14 days post injection. After each injection, participants were seen at 24 hours, 48 hours, day 7 and day 14. At each visit, the injection site was examined and the subjects were asked about adverse events. Additionally, participants were interviewed for any abnormal signs or symptoms on Day 30, 60, 61, 62, 74, 90, 180, 181, 182, 194 and 240 after each vaccination. Blood samples (10–20 mL) for complete blood count (CBC) and differential, measurement of blood creatinine level, AST, ALT, urine sedimentation, were drawn at screening and on Day 0, 30, 60, 90, 180 and 240.

Clinical adverse events (AEs) were classified into local and systemic events. Solicited local adverse events included tenderness, local pain, swelling, induration, nodule, ulceration, itching and erythema. Systemic adverse events included fever, rash, headache, nausea, hypertension, hypotension, and abdominal pain. The intensity of the AEs were graded mild (grade 1; well-tolerated), moderate (grade 2; interfering with daily activities) or severe (grade 3; preventing normal daily activities). Causality relationship to the vaccine was categorized as definite, probable, possible, unlikely or not related.

### Immunogenicity

All immunological analyses was performed according to standard operating procedure (SOP) documents available on-site. All the time-point samples (collected on Day 0, 30, 60, 90, 180, 194 and 240) from participants were analyzed by IFA for determination of antibodies to cultured parasite of P. falciparum FCC1/HN isolate, ELISA for IgG to PfCP-2.9 recombinant protein and lymphocyte proliferation assay for T cell immune response to the antigen. Sera post three immunizations ( on day 194) were tested for their ability to inhibit growth of the parasite in an in vitro growth inhibition assay (GIA).

Responders to the vaccine was defined at two levels: responders were those with ELISA titers specific for PfCP-2.9 protein greater than 1∶500 and high responders were those with titres greater than 1∶10,000. Lymphocyte responder was defined as a participant whose lymphocyte stimulation indices are greater than 3.

### ELISA

ELISA was performed in triplicate on diluted serum samples in 96-well flat-bottomed micro-titer plates. Micro-titer plates (Thermo Lab systems) were coated for 1 h at 37°C with 100 µL of recombinant protein at the concentration of 1.0 µg/mL diluted in carbonate buffer (0.159% Na_2_CO3, 0.293% NaHCO3, pH 9.6). The antigen solution was removed and the plates were blocked with PBS containing 3% skim milk at 37°C for 1 h. 100 µL of serum samples at various dilutions were added to the plates, and incubated at 37°C for 1 h. 100 µL of peroxidase-conjugated horse anti-human IgG were added to the plate at 1∶1000 dilution in PBS containing 3% skim milk for a further 1 h incubation at 37°C. For every step, plates were washed three times with PBST (PBS containing 0.05% Tween 20) and one time with PBS using a plate washer. Bound secondary antibodies were detected by adding 100 µL of TMB (3,3′, 5,5′-Tetramethyl Benzidine) substrate solution at room temperature for 10 min, and the reaction was stopped by adding 50 µL of 2 M H_2_SO4. The plates were read at an absorbance of 450 nm (ELx800 ELISA reader, BIO-TEC Instrument INC). Cutoff values were determined as the mean plus three standard deviations for the pre-immune sera.

### IFA

IFA was performed as previously described [22]. Cultured parasites of Pf FCC1/HN isolate were used as antigens. IFA slides were prepared using culture material with a 5%–10% parasitemia. The slides were blocked with 3% nonfat powdered milk in PBS before adding serum samples at various dilutions and incubated for 1 h at room temperature. After being extensively washed with PBS, the slides were incubated with fluorescein isothiocyanate-labeled secondary Ab of anti-human IgG at 1∶100 with blocking buffer for 1 h at room temperature. The bound secondary antibodies were examined by fluorescence microscope. End-point titers were determined as the last dilution above the background that fluorescent parasites were observed in pre-immune sera.

### Proliferative T cell assay

T-cell responses to the antigen were measured by a lymphocyte proliferation assay. Mononuclear cells from the peripheral blood obtained from the participants were isolated by Ficoll-Hypaque density gradient centrifugation. The collected mononuclear cell were diluted to a concentration of 1×106/mL in complete RPMI 1640 containing 10% FCS, 10 mmol/L Hepes, 1 mmol/L sodium pyruvate, 2 mmol/L glutamine, 0.075%NaHCO3, 100 µg/mL streptomycin, and 100u/mL penicillin. 100 µL of the cell suspension and 100 µL medium with or without the vaccine antigen were dispensed into 96-well plate in triplicate. After culture at 37°, 5%CO2 for 60 h, the culture were pulsed with 1 µci of [3H]TdR for 12 h before harvesting the cells using a semi-automated sample harvester. Its incorporation was determined by β-scintillation counter. Results were expressed as fold increases in stimulation index (SI), calculated as post-vaccination SI divided by pre-immune SI.

### Growth Inhibition Assay

The immune sera post three immunizations ( on day 194) were tested for their ability to inhibit growth of the parasite *in vitro*. *Plasmodium falciparum* FCC1/HN parasite line was used for the assay. FCC1/HN line was isolated from Hainan Island, China and its continuous cultivation *in vitro* was established in 1979 [22]. The parasite culture with a majority of rings was synchronized by treatment with 5% sorbital. The inhibition assay was performed with the starting culture containing 2% hematocrit and approximately 0.3% parasitaemia using the synchronized parasite with mostly late-trophozoites and schizonts. 170 µL of the culture suspension and 30 µL of the test serum were added in triplicate wells to 96-well flat-bottomed plates and incubated at 37°C for 72 h. Thin blood smears were prepared to determine parasitaemia. The inhibition rate was determined according to the following: %inhibition = [(1-(Pt-Ps)/(Pc-Ps) ]×100%. Pc: parasitemia of pre-immune sera; Pt: parasitemia of immune sera; Ps: parasitemia of starting culture.

#### Sample size

The sample size for this study was based on the primary objective of evaluating safety of each dose level. The study was not powered to detect statistically significant differences in the frequency of adverse events or immunogenicity between different study arms. The number of subjects in each study arm (10) is a generally acceptable sample size in a Phase I study for detecting large differences in the frequency of adverse events with reasonable accuracy.

#### Randomization

Two numbers were generated for each volunteer in the study, a *screening number* and a *randomization number*. The screening number was given to the participants at the first screening visit (composed of 3 digits starting with 001 and given in a sequential fashion).

Randomization numbers composed of 3 digits starting with 801 and specifying a treatment code were generated by the data management team and were randomly assigned to the eligible participants meeting all inclusion and none of the exclusion criteria, prior to vaccination on Day 0. The initials, screening number and the randomization number will be written by the vaccine injector on the “Vaccine Information and Administration Form” accompanying each treatment vial.

### Blinding

The administration of the vaccine was single-blinded in that study participants were blinded to the vaccine assignment. Only the study pharmacists had access to the randomization codes that revealed the dose group allocation. However the investigators and outcome assessors were not blinded to the dose group allocation.

### Statistical methods

Descriptive statistics were used to analyse the study data. Local and systemic events were summarized as frequency tables of numbers of adverse events by vaccination sequence, study arm and intensity of reported event. Immunogenicity data was summarized by vaccine group as geometric mean titres (GMTs) and the associated 95% confidence interval if required. Mixed models were used to analyse immunogenicity parameters and laboratory tests among groups over time. If the mixed models analysis showed a significant difference between groups, Fisher's LSD was used to analyse the difference between any two groups at each time point. All statistical analyses tests were performed at an individual significance level of 5% and were two-sided and regarded as exploratory. SAS 8.2 was used for the statistical analyses.

## Results

### Participant flow, recruitment and baseline data

Prior to starting the study, workers in factories, students in universities and residents in Shanghai were invited to attend an information meeting to describe the clinical trial. Within a month of scheduled enrollment, all individuals who agreed to participate from the previously held information meeting were invited to consent for the study. Inclusion and exclusion criteria were checked and the participants were provided with a screening number (3 digits in a sequential fashion). The study physicians took the clinical history and conducted a physical examination for screening purposes.

**Figure 1:**
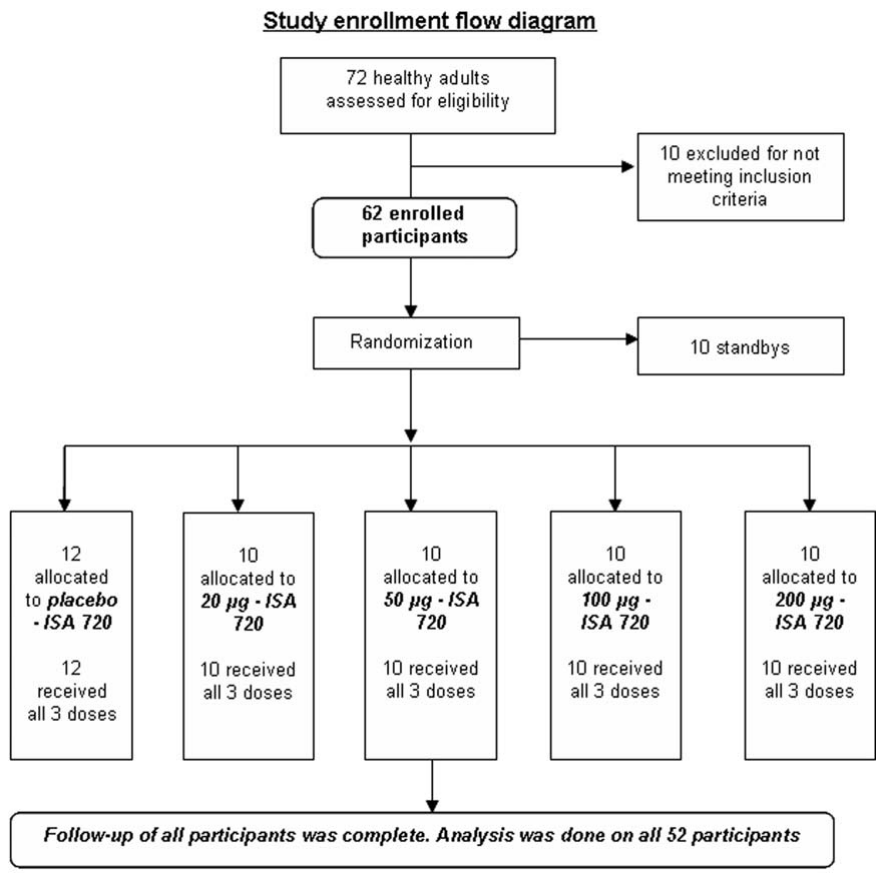
Study enrollment flow diagram.

Seventy-two participants were screened according to the inclusion and exclusion criteria, and 62 participants were enrolled (Fig 1). Fifty-two participants were enrolled as participants and randomly allocated to 4 vaccine groups (10 per group receiving 20 µg, 50 µg, 100 µg or 200 µg doses) and one placebo group of 12. The remaining 10 participants were enrolled as standbys in case any withdrawals occurred before the first vaccination. No participants were withdrawn or removed during the study from August 11, 2003 through November 18, 2004, and all received the three scheduled vaccinations. The numbers analyzed include all 52 participants. The 52 participants, 32 males and 20 females, were all Chinese with ages ranging from 18 to 45 and a mean age of 24.5.

### Outcomes and estimation

#### Safety and reactogenicity

Local adverse events observed during the trial included tenderness, pain, swelling, induration, erythema or redness and itching. All local AEs related to the vaccination resolved within 1 to 2 weeks without treatment. Neither vaccine nor placebo recipients experienced any grade 3 or severe reactions.

The most common adverse event reported was tenderness (58%). This was followed by swelling (18%), induration (9%) and pain at the injection site (10%). 93% of these events were graded as grade 1 or mild in intensity. 75% of the vaccinees experienced tenderness , followed by swelling (42%), local pain (25%), induration(23%), itching (8%), and redness at the injection site (5%). 58% of participants in the placebo control group compared with 80% of total participants in the vaccine groups experienced at least one local adverse event (Table 1). All the events in the placebo group were graded as mild. Out of those vaccinated, 65% experienced events that were graded mild and 15% (6 subjects) experienced moderate adverse events. There was a trend towards more adverse reactions and slightly more moderate adverse reactions with increasing vaccine dose and number of immunizations (Table 2). 8 events of grade 2 or moderate intensity that occurred in the six participants included induration (4 events) and swelling (2 events). 5 were from the highest dose cohort (200) and one from the lowest dose cohort(20). The moderate AE (swelling) in the volunteer receiving 20 dose followed the second immunization.

**Table 1:** Number (percentage) of subjects experiencing solicited adverse events by study arms and intensity level.

Study Arm (N)	*ISA 720/Control (N = 12)*				*PfCP-2.9/ISA 720 (20* µ*g) (N = 10)*				*PfCP-2.9/ISA 720 (50* µ*g) (N = 10)*				*PfCP-2.9/ISA 720 (100* µ*g) (N = 10)*				*PfCP-2.9/ISA 720 (200* µ*g) (N = 10)*			
Intensity	*Any*	*G1*	*G2*	*G3*	*Any*	*G1*	*G2*	*G3*	*Any*	*G1*	*G2*	*G3*	*Any*	*G1*	*G2*	*G3*	*Any*	*G1*	*G2*	*G3*
Any, n (%)	12(100)	12	1	0	10(100)	10(100)	3	0	10(100)	10(100)	1(10)	0	9(90)	9	0	0	10 (100)	10	3	0
***Local***	7(58)	7	0	0	6(60)	6	2	0	8(80)	8	1	0	9(90)	9	0	0	9(90)	9	3	0
Tenderness	7(58)	7	0	0	4(40)	4	0	0	8(80)	7	1	0	8(80)	8	0	0	9(90)	9	0	0
Pain	0	0	0	0	6(60)	5	1	0	0	0	0	0	2(20)	2	0	0	2(20)	2	0	0
Swelling	0	0	0	0	3(30)	2	1	0	3(30)	3	0	0	3(30)	3	0	0	8(80)	7	1	0
Induration	0	0	0	0	0	0	0	0	2(20)	2	0	0	2(20)	2	0	0	5(50)	2	3	0
Erythema	0	0	0	0	0	0	0	0	0	0	0	0	0	0	0	0	2(20)	2	0	0
Itching	0	0	0	0	1(10)	1	0	0	0	0	0	0	1(10)	1	0	0	1(10)	1	0	0
***General***	8(67)	8	1	0	9(90)	8	2	0	10(100)	10	0	0	8(80)	8	0	0	8(80)	8	1	0
Fever	0	0	0	0	0	0	0	0	0	0	0	0	1(10)	1	0	0	1(10)	0	1	0
Rash	0	0	0	0	2(20)	1	1	0	0	0	0	0	0	0	0	0	0	0	0	0
Headache	0	0	0	0	1(10)	1	0	0	0	0	0	0	0	0	0	0	0	0	0	0
Hypertension	1(8)	1	0	0	4(40)	4	0	0	2(20)	2	0	0	2(20)	2	0	0	0	0	0	0
Hypotension	10(83)	10	0	0	8(80)	7	1	0	10(100)	10	0	0	6(60)	6	0	0	7(70)	7	0	0
Diarrhea	0	0	0	0	0	0	0	0	0	0	0	0	0	0	0	0	2(20)	1	1	0
Abdominal pain	0	0	0	0	0	0	0	0	0	0	0	0	0	0	0	0	1(10)	1	0	0

**Note:** N represents the number of participants randomized into each study arm, receiving at least one vaccination. Denominators are participants receiving at least one vaccination for each study arm and the numerators are the number of volunteers experiencing the event per immunization. Each participant is counted at the most once for each event type per vaccination and, the greatest reported intensity level is recorded.

**Table 2:** Number (percentage) of subjects experiencing local solicited adverse events for each study arm receiving vaccine, by intensity grade for each immunization.

Immunization schedule	*First immunization (N = 10)*				*Second immunization (N = 10)*				*Third immunization (N = 10)*			
Intensity	*Any*	*G1*	*G2*	*G3*	*Any*	*G1*	*G2*	*G3*	*Any*	*G1*	*G2*	*G3*
***20*** ** µ** ***g***												
Tenderness	1(10)	1(10)	0	0	0	0	0	0	4(40)	4(40)	0	0
Pain	0	0	0	0	6(60)	5(50)	1(10)	0	1(10)	1(10)	0	0
Swelling	0	0	0	0	2(20)	1(10)	1(10)	0	2(20)	2(20)	0	0
Induration	0	0	0	0	0	0	0	0	0	0	0	0
Erythema	0	0	0	0	0	0	0	0	0	0	0	0
Itching	0	0	0	0	0	0	0	0	1(10)	1(10)	0	0
***50*** ** µ** ***g***												
Tenderness	1(10)	1(10)	0	0	5(50)	4(40)	1(10)	0	4(40)	4(40)	0	0
Pain	0	0	0	0	0	0	0	0	0	0	0	0
Swelling	1(10)	1(10)	0	0	0	0	0	0	2(20)	2(20)	0	0
Induration	2(20)	2(20)	0	0	0	0	0	0	0	0	0	0
Erythema	0	0	0	0	0	0	0	0	0	0	0	0
Itching	0	0	0	0	0	0	0	0	0	0	0	0
***100*** ** µ** ***g***												
Tenderness	4(40)	4(40)	0	0	6(60)	6(60)	0	0	5(50)	5(50)	0	0
Pain	2(20)	2(20)	0	0	0	0	0	0	0	0	0	0
Swelling	0	0	0	0	0	0	0	0	3	3	0	0
Induration	1(10)	1(10)	0	0	1(10)	1(10)	0	0	0	0	0	0
Erythema	0	0	0	0	0	0	0	0	0	0	0	0
Itching	1(10)	1(10)	0	0	0	0	0	0	0	0	0	0
***200*** ** µ** ***g***												
Tenderness	7(70)	7(70)	0	0	6(60)	6(60)	0	0	8(80)	8(80)	0	0
Pain	1(10)	1(10)	0	0	1(10)	1(10)	0	0	0	0	0	0
Swelling	1(10)	1(10)	0	0	2(20)	2(20)	0	0	8(80)	7(70)	1(10)	0
Induration	4(40)	1(10)	3	0	1(10)	1(10)	1(10)	0	2(20)	1(10)	1(10)	0
Erythema	0	0	0	0	0	0	0	0	2(20)	2(20)	0	0
Itching	0	0	0	0	0	0	0	0	1(10)	1(10)	0	0

**Note:** N represents the number of participants randomized into each study arm, receiving at least one immunization. Denominators are participants receiving at least one immunization for each study arm. Each participant is counted at the most once for each event type per vaccination and the greatest reported intensity level is recorded. For the control group, the only observed event reported was mild tenderness (grade 1); 3 occurred following first immunization; 4 after the second immunization and 5 following the third immunization.

17 vaccinees developed swelling, 30% of the 20 µg, 50 µg , and 100 µg respectively and 80% of the vaccinees in the 200 µg group (Table 3). All except two were graded mild.

**Table 3:** Characteristics and evolution of swelling occurring following immunization occurring in the study.

*No per dose group*	*Gender*	*Immunization*	*Intensity*	*Duration (days)*	*Resolution*
***20 µg***					
1	Male	Second	Grade 1	7	spontaneous
		Third	Grade 1	5	
2	Male	Second	Grade 2	3	spontaneous
3	Female	Third	Grade 1	2	spontaneous
***50 µg***					
1	Male	First	Grade 1	2	spontaneous
2	Female	Third	Grade 1	4	spontaneous
3	Male	Third	Grade 1	2	spontaneous
***100 µg***					
1	Female	Third	Grade 1	6	spontaneous
2	Female	Third	Grade 1	2	spontaneous
3	Female	Third	Grade 1	6	spontaneous
***200 µg***					
1	Female	First	Grade 1	4	spontaneous
		Second	Grade 1	10	
		Third	Grade 1	7	
2	Female	Second	Grade 1	5	spontaneous
		Third	Grade 1	5	
3	Male	Third	Grade 1	3	spontaneous
4	Male	Third	Grade 2	3	spontaneous
5	Male	Third	Grade 1	3	spontaneous
6	Female	Third	Grade 1	4	spontaneous
7	Female	Third	Grade 1	3	spontaneous
8	Male	Third	Grade 1	3	spontaneous

Discrepancies in defining a nodule versus induration due to differences in the terminology used in Mandarin versus English resulted in some difficulties in interpretation of the diagnosis of suspected nodules. For example, the term ‘knot’ is a literal translation of the Mandarin term used to describe some of the observations suspected as nodules, but it was not possible to fully define and conclude if it was distinguished from induration or swelling in all cases. As a result, all suspected cases of nodules were classified under induration as shown in Table 4. A total of 9 participants developed mild to moderate induration within 2 to 4 weeks post-vaccination (delayed reaction) in the 50 µg (20%) , 100 µg (10%) and 200 µg (60%) vaccine groups, whereas induration was not observed in participants receiving 20 µg of the vaccine antigen or the placebo (Table 4). Moreover, only participants (3) receiving the highest dose of the vaccine (200 µg) developed induration graded as moderate. The size of the induration ranged from less than 2 cm to up to 4 cm in diameter. These data suggest that development of induration and its severity is likely related to the vaccine formulation and is dose-dependent. More participants receiving the highest vaccine dose experienced swelling and induration than those in the lower dose cohorts. In addition, no AEs apart from tenderness were observed in the placebo group. Therefore, these adverse events were likely related to the vaccine.

**Table 4:** Characteristics and evolution of induration* following immunization occurring in the study.

*No per dose group*	*Gender*	*Immunization*	*Intensity*	*Nodule-like features*	*Duration* *(days)*	*Resolution*
***50 µg***						
1	Female	First	Grade 1	‘knot’ at injection site 18 days post-immunization	9	spontaneous
2	Female	First	Grade 1		5	spontaneous
***100 µg***						
1	Female	Second	Grade 1	‘knot’ at injection site 13 days post-immunization	13	spontaneous
***200 µg***						
1	Male	First	Grade 2		5	spontaneous
2	Female	First	Grade 1	‘knot’ at injection site 14 days post-immunization	6	spontaneous
3	Female	First	Grade 2		4	spontaneous
4	Female	First	Grade 2	‘knot’ at injection site 15 days post-immunization	4	spontaneous
5	Male	Second	Grade 1	‘knot’ at injection site 15 days post-immunization	6	spontaneous
		Third	Grade 2		8	spontaneous
6	Male	Third	Grade 1	‘knot’ at injection site 15 days post-immunization	7	spontaneous

* **Note:** Due to discrepancies in the defining and diagnosing nodules versus induration, all suspected nodules were classified as induration. The word ‘knot’ is a literal translation of the mandarin word used to describe the AE.

There was no induration reported in the control or 20 µg dose groups.

Systemic adverse reactions observed in this study include fever, rash, headache, hypotension and hypertension, abdominal pain, diarrhea, and the common cold. Hypotension (78%) and hypertension (20%) were the most frequently reported (Table 1), occurring at a similar rate in the placebo control group [(10/12)100 = 83%] as well as in the vaccine groups [(31/40)100 = 78%]. All the events were graded as mild, except for in one participant, in which it was grade as moderate. All were asymptomatic and resolved spontaneously. Moreover, the events were similar for participants receiving the vaccine or placebo, and there was no trend towards increased reactions with increasing doses of the vaccine. It is worth noting here that in the protocol, hypertension after vaccination was defined as diastolic pressures transiently increasing >10 mmHg compared to the blood pressure measured before injection, while hypotension was defined as a systolic pressure decrease of >10 mm Hg. This is likely more rigorous than that of standard clinical practice and likely contributed to the high rates of hypotension and hypertension. The other systemic adverse events, including fever in 2, rash in 1, headache in 1, diarrhea in 2 and common cold in 4 participants were judged to be not related to vaccination.

Results from the mixed model analysis of laboratory tests indicated that no significant differences (P>0.05, Fisher tests of fixed effects) were observed between vaccine groups and placebo group as well as between different time-point samples. Four participants were reported to have a mild decrease in white blood cell account on day 90 and one volunteer to have a mild elevation in ALT and AST on Day 30, but the values returned to normal without therapy and judged to be unrelated to vaccination.

During the trial, no serious adverse events or unexplained illnesses occurred in the participants. One volunteer in the placebo group suffered from acute appendicitis after the second injection and was operated on. The volunteer received the third injection following his recovery. The event was judged not to be related to the vaccination.

#### Immunogenicity

##### 

**ELISA titers:** Antibodies specific to the PfCP-2.9 vaccine antigen were measured by ELISA. A specific antibody response (titer >1∶200) was detected in all the participants receiving the vaccine injection, whereas no specific antibody (titer <1∶200) was detected in the placebo group. Depending on the post-immunization serum antibody levels to PfCP-2.9, participants were labeled either “antibody responder” (titer >1∶500) or “high antibody responder” (titer >1∶10,000). According to this criteria, almost all participants receiving the vaccine developed positive antibody responses to PfCP-2.9 after the first immunization. Only 2 participants in the 50 µg group and 1 in the 200 µg group had titers below 1∶500 (negative response) (Table 5, 6 and 7). After the second vaccination, the antigen-specific antibody levels in all groups significantly increased. However, these levels declined 4 months later and 4 participants became negative (titer <1∶500). After the third vaccination, antibody levels in all participants were boosted and by day 240, the last study day, all participants had positive antibody responses. The geometric mean ELISA titers were 15,193 in the 20 µg, 9,666 in the 50 µg, 9, 642 in the 100 µg and 10,530 in 200 µg vaccine dose groups (Table 5, 6 and 7).

**Table 5:** ELISA antibody titer in time-point serum samples from participants in 20 and 50 µg groups.

Dose (µg)	No. participants	ELISA titres			
		Day30	Day 90	Day194	Day240
20	1	4,758	22,450	45,897	38,519
	2	3,858	12,732	7,556	25,325
	3	2,188	2,188	50,310	13,544
	4	1,410	8,546	5,434	5,722
	5	966	27,684	8,488	7,906
	6	1,811	12,996	7,396	6,832
	7	1,029	23,877	15,641	15,805
	8	3,939	20,636	16,097	18,754
	9	4,189	9,297	14,388	11,225
	10	10,539	42,100	28,524	48,254
**Geomean**		**2,660**	**14,352**	**15,097**	**15194**
50	1	1,178	10,943	9,356	8,509
	2	973	11,732	11,071	9,018
	3	478	2,346	2,192	1,375
	4	4,265	22,826	69,372	24,802
	5	4,024	9,915	9,428	3,160
	6	5,266	15,835	24,802	31,462
	7	2,192	32,025	11,462	17,689
	8	8,186	11,178	53,064	28,515
	9	579	4,433	6,759	6,136
	10	8,676	9,018	7,026	8,845
**Geomean**		**2,317**	**10,444**	**12,667**	**9,666**

**Note:** A: ELISA antibody titer to the PfCP-2.9 in pre-immune serum samples(Day 0) is <1∶100

B: ELISA antibody titer to the PfCP-2.9 in sera from participants receiving the placebo injection is <1∶100

C. The first vaccination was performed on day 0 and boosted vaccination were on days of 60 and 180

D. ND: not done

**Table 6:** ELISA antibody titer in time-point serum samples from participants in 100 and 200 µg groups.

Dose (µg)	No. participants	ELISA titres			
		Day30	Day 90	Day194	Day240
**100**	1	902	7,725	6,759	5,690
	2	2,419	11,071	21,373	18,920
	3	5,790	20,964	41,423	108,568
	4	3,653	3,798	12,849	11,049
	5	6,502	3,259	11,157	10,733
	6	3,798	2,097	20,246	5,369
	7	2,808	18,167	26,907	11,869
	8	1,272	2,017	2,171	2,171
	9	4,025	4,348	34,269	7,725
	10	1,455	4,025	4,183	4,689
**Geomean**		**2,740**	**5,591**	**13,070**	**9,642**
**200**	1	318	2,012	2,269	1,833
	2	3,850	111,274	71,096	74,079
	3	12,864	37,370	74,079	37,140
	4	1,216	10,539	23,391	25,447
	5	667	3,840	3,619	2,143
	6	1,891	11,003	13,294	11,004
	7	1,295	9,908	12,473	7,074
	8	5,061	11,465	28,259	28,846
	9	345	1,023	1,149	1,764
	10	2,698	15,701	18,205	ND
**Geomean**		**1648**	**9621**	**12579**	**12579**

**Note:** A: ELISA antibody titer to the PfCP-2.9 in pre-immune serum samples(Day 0) is <1∶100

B: ELISA antibody titer to the PfCP-2.9 in sera from participants receiving the placebo injection is <1∶100

C. The first vaccination was performed on day 0 and boosted vaccination were on days of 60 and 180

D. ND: not done

**Table 7:** Geometric mean ELISA titers and seroconversion.

Groups	Seroconversion*		GMT (95% CI)***	Titers	
	n**	%		Minimum	Maximum
**20 µg**					
Day 30	10	100	2660 (1540, 4593)	966	10539
Day 60	10	100	3010 (1721, 5266)	870	19362
Day 90	10	100	14352( 7926, 25989)	2188	42100
Day 180	9	90	2734(1430, 5228)	464	18493
Day 194	10	100	15097 (8647, 26358)	5434	50310
Day 240	10	100	15194 (9095, 25383)	5722	48254
**50 µg**					
Day 30	9	90	2317 (1077, 4984)	478	8676
Day 60	10	100	2420 (1232, 4754)	500	8509
Day 90	10	100	10444(6112, 17848)	2346	32025
Day 180	10	100	2729 (1492, 4994)	743	17433
Day 194	10	100	12668(6093, 26336)	2192	69372
Day 240	10	100	9666(4707, 19849)	1375	31462
**100 µg**					
Day 30	10	100	2740 (1713, 4384)	902	6502
Day 60	10	100	2457 (1602, 3768)	1031	9538
Day 90	10	100	5591 (3070, 10182)	2017	20964
Day 180	9	90	1551 ( 728, 3307)	254	10630
Day 194	10	100	13070(6604, 25866)	2171	41423
Day 240	10	100	9642 (4570, 20343)	2171	108568
**200 µg**					
Day 30	8	80	1648 ( 708, 3839)	318	12864
Day 60	8	80	1227 ( 619, 2436)	331	5472
Day 90	10	100	9621 (3658, 25306)	1023	111274
Day 180	7	70	2145( 904, 5085)	365	9970
Day 194	10	100	12579 (4634, 34142)	1149	74079
Day 240	10	100	10531 (4024, 27559)	1764	74079

**Note:**

*: ELISA titer ≥1∶500

**: Number of participants in individual group

***: Geometric mean titers (GMTs) are calculated by exponentiating the mean of the log-transformed values.

The frequencies of high responders were as follows: 90% (9/10) in the 20 µg group after the second immunization but decreased to 60% (5/10) after the third immunization; in the 50 µg group, 60% after the second and 50% after the third vaccinations; in the 100 µg group, 30% after the second but increased to 60% after the third vaccination; and 60% after the second and 70% after the third vaccinations in the 200 µg group. No relationship was found between antigen doses and frequencies of the high antibody responders after the second and third vaccinations. The third vaccination did not increase frequencies of high responders in the 20 µg and 50 µg groups, but it did so in the remaining two vaccine groups.

A mixed model analysis of ELISA titers indicated that the overall group effect was significant (Table 8; P<0.001). In comparing antibody levels in participants receiving different doses of the antigen, there were no significant dose-response relationships among the 20 µg, 50 µg, 100 µg and 200 µg groups on day 30 (after the first vaccination), day 90 (after the second vaccination), day 194 (after the third vaccination) and on the last study day (day 240) (P>0.05, ANOVA/LSD). One exception was in the comparison between the 20 µg and 100 µg groups on day 90 in which the geometric mean titer of 14,352 in the 20 µg group was significant higher than that of 5,591 in the 100 µg group.

**Table 8:** Summary of mixed model analysis of ELISA, IFA and the Lymphocyte Stimulation Test (LST).

Fisher tests of fixed effects	F-value			P-value		
	ELISA	IFA	LST	ELISA	IFA	LST
Overall group effect	49.83	6.84	1.90	<0.0001	0.0002	0.126
Interaction of group and collection time	6.19	3.39	4.26	0.0004	0.0162	0.005

**Note:** Models use the log-transformed titer values.

##### 

**Figure 2:**
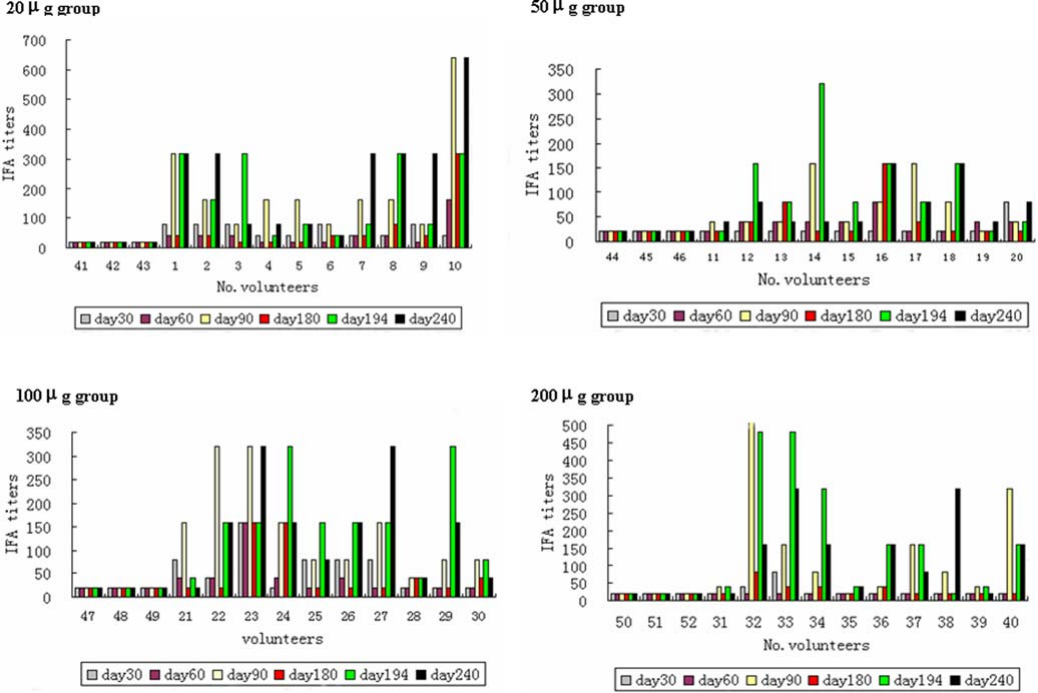
Antibody response to the cultured parasite measured by IFA in sera from participants in groups receiving 20 µg, 50 µg, 100 µg and 200 µg of the test vaccine formulation and the placebo, respectively. The IFA titers in sera collected from the days 30, 60, 80, 180, 194 and 240 were indicated by different symbols. The x-axis is the volunteer's consecutive number: No.1∼10 receiving 20 µg, No.11∼20 receiving 50 µg, No.21∼30 receiving 1000 µg, and No.31∼40 receiving 200 µg of the vaccine while No.41∼52 receiving the placebo.

**Figure 3:**
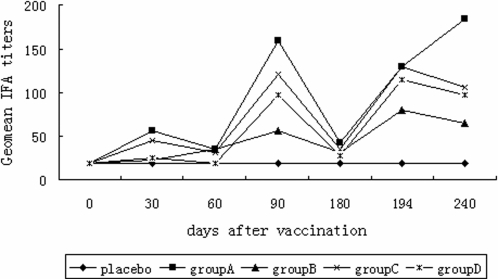
Kinetics of geometric mean values of the IFA titers to the parasite for the groups receiving 20 µg(A, ▪), 50 µg(B,Δ), 100 µg(C, ×) and 200 µg(D, *) of the test vaccine formulation and the placbo(♦). The value was shown at the time-point days 0, 30, 60, 90,180,194 and 240.

**IFA titers:** To determine whether the antigen-specific antibodies from participants recognized native parasite proteins, we used an *in vitro* cultured FCC1/HN strain of P. falciparum as antigen to detect interactions of the serum samples from various time-points (Day 0, 30, 60, 90, 180,194 and 240) with the parasite in an immunofluorescence assay (IFA). As shown in Figure 2, 95% after the second or 92.5% after the third vaccination of participants receiving the vaccine formulation developed a detectable antibody response to the parasite, and the antibody levels were significantly increased after each boost vaccination (Fig. 3). In contrast, both pre-immune sera and sera from participants receiving the placebo had no reactivity to the parasite. A comparison of geometric mean antibody titers showed no relationship between vaccine dose and antibody level. The lowest dose (20 µg) of the antigen generated the highest antibody response to the parasite after the second immunization as well as on the last samples, while the 50 µg dose gave the lowest antibody response to the parasite (Fig. 3). The IFA antibody titers were consistent with the ELISA titers to the recombinant protein in most samples. For example, a subject in the 200 µg group who had the highest ELISA titer at 1∶111,274 also had the highest IFA titer at 1∶1,280 (Fig. 2).

##### 

**Figure 4:**
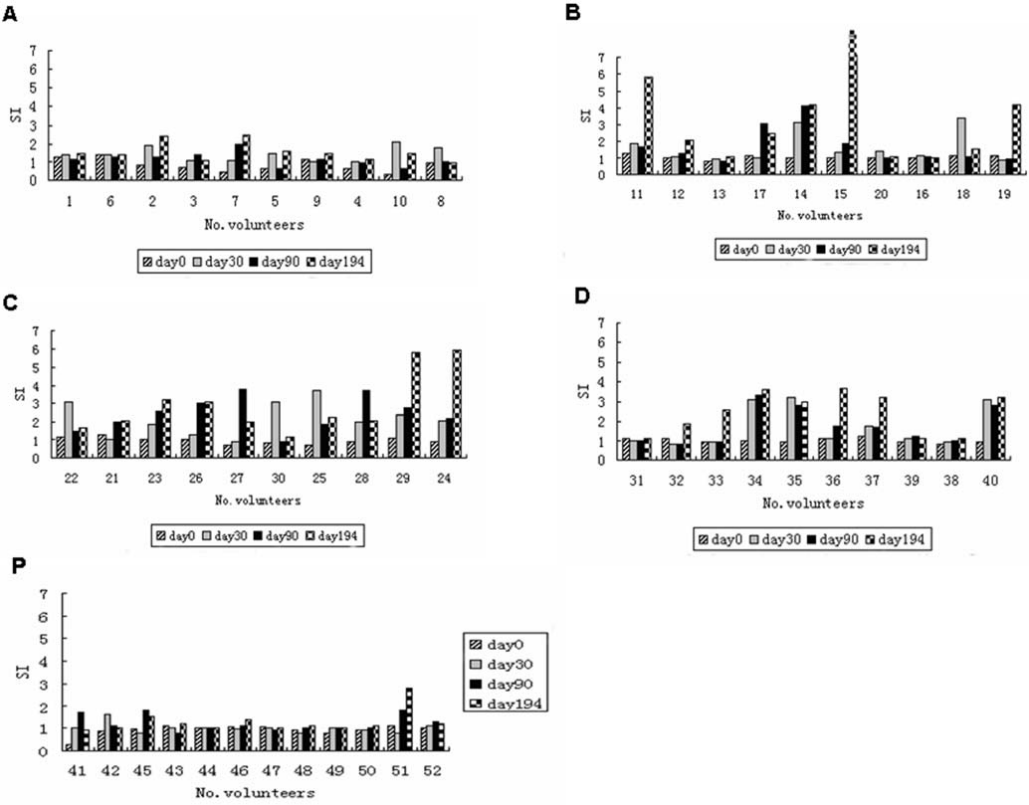
T-cell response to the PfCP-2.9 protein by Lymphocyte Proliferation Assay. Peripheral blood mononuclear cells were separated from blood of participants receiving either the vaccine formulation (group A to D) or the placeb(group P) on the days 0, 30, 90 and 194 and tested for their proliferation. The results were expressed as the stimulation index (SI), SI = mean cpm of culture with the antigen/mean cpm of culture without the antigen.

**T-cell response:** In contrast to the high antibody response, the antigen did not induce correspondingly strong T-cell responses. Only 50% (20/40) of the participants receiving the vaccine formulation had a positive T-cell response (SI>3). As shown in Figure 4, after the first vaccination (day 30) no participant had a positive T-cell response in the lowest dose group (20 µg) while positive T-cell responses were induced 2 in the 50 µg group, 3 in the 100 µg group and 3 in the 200 µg group, suggesting higher dose of the vaccine required to induce the T cell response.

##### 

**In vitro growth inhibition assay (GIA):** Post- immunization sera at 15% concentration did not show any significant inhibition of parasite growth compared to the pre-immune sera (data not shown).

## Discussion

### Interpretation

The primary objective of the study was to assess the safety and reactogenicity of the PfCP-2.9 vaccine candidate formulated with the Montanide ISA720 adjuvant in a randomized, single blinded, placebo-controlled single center clinical trial. The results demonstrated that the four dose levels of PfCP-2.9/ISA720 vaccine formulation were safe and well-tolerated in Chinese adult malaria-naïve participants. All 52 participants completed three vaccinations and all follow-ups with no withdrawals or removals. Throughout this trial, there were no severe or serious adverse events related to the vaccinations.

High frequencies of local reactions to the vaccinations were observed in this study, but the majority of events were graded as mild. 6 participants developed moderate local adverse events. The rapidly occurring local adverse events such as tenderness, pain and swelling were observed after injections and mostly disappeared within 1 week. The delayed reaction, injection site induration (including suspected nodules), occurred 2 to 4 weeks following vaccination and lasted from 4 to 13 days. These observations were similar to that in other studies using the Montanide ISA720 adjuvant in humans where the delayed reactions developed only in those participants receiving the antigen [23], [24]. Moreover, the frequency of the adverse reactions was higher in the participants receiving high doses than those in low doses, and moderate induration developed only in the participants who received the highest dose of the vaccine (200 µg). These results indicate that the generation of induration and/or nodule and its severity were antigen- as well as dose-dependent.

None of the systemic adverse events observed in this study were judged to be related to vaccination. There was a high rate of hypotension and hypertension observed in the trial. However, this was equally distributed between participants in the placebo group and those vaccinated and did not show any relationship with increasing vaccine dose or number of immunization. In addition, the cut-offs for categorizing a reading of hypotension or hypertension was more rigorous that what is normal clinical practice. These reasons were considered to be the most likely explanation for the high frequencies of hypotension and hypertension observed in this study. Moreover, all cases of hypotension and hypertension were asymptomatic and no treatment was required. Throughout the trial, 4 participants experienced the common cold and another 6 experienced fever, rash, headache and diarrhea. These systemic AEs were judged to be unrelated to the vaccination.

The results of the immunogenicity evaluation of the PfCP2.9 vaccine showed that antigen specific antibody responses were induced in the participants receiving the test vaccine. Of 40 participants receiving various doses of the test antigen, 38 had antigen-specific antibodies with ELISA titers of greater than 1∶500 (responder) after the first vaccination. Sixty-percent of vaccinated participants had an antibody titer of more than 1∶10,000 (high responder). Importantly, these antibodies recognized native proteins on the cultured parasite measured by IFA, and the ELISA titers were generally consistent with IFA titers. Our results were consistent with the immunogenicity of the chimeric protein in animals.

Immune sera from vaccinated volunteers at this concentration did not significantly inhibit growth of cultured parasites in an in vitro growth inhibition assay (GIA). This was in contrast to our previous data in rabbits and monkeys showed that 15% immune sera almost completely inhibited parasite growth in vitro [22]. A likely explanation could be that a much lower level of specific antibodies are induced in humans compared to that in animals. In a previous study of five monkeys receiving the vaccine formulation, strong immune response were generated to the vaccine antigen with ELISA end titers of 1/867,000 to 1/5,700,000 and IFA end titers of 1/2,560 to 1/40,960 [22], whereas in human the ELISA as well as IFA titers of the specific antibodies were hundreds of times lower than that in the animals. We noted only one report on Phase I clinical trial showing that purified IgG from volunteers vaccinated with PfAMA1 had a certain level of parasite growth inhibition with 4 of 22 sera tested at inhibition rate of 14, 17, 18 and 50% against the FVO isolate, respectively [23]. Further experiments could be considered for this vaccine candidate to test total IgG or affinity-purified antibodies from vaccinated volunteers for their biological activities.

To identify the optimal dose of PfCP-2.9 vaccine and immunization regimens in humans, 4 different vaccine dose regimens were tested in the current study. A mixed model analysis of ELISA titers indicated that the overall group effect was significant (P<0.001). However, ANOVA/LSD analysis of ELISA titers between the 4 dose groups showed no significant difference in geometric mean ELISA titers (P>0.05) on day 30 (post first immunization), day 90 (post second immunization) and day 194 (post third immunization), except one comparison between the 20 µg and 100 µg groups on day 90. Therefore, no evidence of significant dose-response relationship was observed and vaccine doses higher than 20 µg may not be necessary. Vaccine doses below 20 µg could be considered to identify the optimal dose in future clinical studies.

The antibody response in all vaccine groups was efficiently boosted after the second immunization. However, in comparing the ELISA titers between days 90 and 194, there was no significant difference in geometric mean titers (P>0.05) in the 4 dose groups, indicating that the third boosting immunization (day 180) did not significantly increase the antibody levels in all groups.

### Generalizability

The study sample size was designed to be able to detect large differences in the frequency of adverse events between the dose groups. These results demonstrating safety and tolerability of PfCP2.9 formulated with ISA 720 in healthy Chinese adults who are malaria naive are however not generalizable to the main target population of the vaccine, children at risk of malaria. Further clinical trials will be in such target populations will eventually be required in order to obtain generalizable data.

The vaccine promisingly demonstrates immunogenicity in all the dose ranges tested. However, complex interactions between the host and parasite under conditions of natural malaria exposure, and similarly, the impact of such interactions on vaccine induced immune responses can only be further explored in trials in settings of natural exposure in the target population. Immunogenicity in the face of pre-existing immunity and natural boosting will have to be assessed to make more generalizable conclusions.

### Overall evidence

Development of a malaria vaccine against the asexual stage is attractive because only this stage of the parasite is known to cause disease and even death. Antibodies against merozoites are believed to be essential for blocking the parasite invasion of host erythrocytes [25]. IgG prepared from individuals in malaria-endemic regions can reduce parasitemia in the malaria-infected patient [26]. Moreover, maternal antibodies passively transferred to the fetus provided protection against clinical malaria [27]. P. falciparum MSP1 and AMA-1 are the two leading asexual blood-stage vaccine antigens [28] because the molecules may be involved in the process of merozoite invasion of erythrocytes. The C-terminal region of MSP1-19 and AMA-1 (III) are thought to be targets of inhibitory antibodies [13], [20]and thus were selected for the construction of this chimeric protein. The PfCP-2.9 protein generated by fusion of the MSP1-19 and AMA-1 (III) was demonstrated to be highly immunogenic in various animal models and the antibodies to this chimeric protein recognized native parasite proteins and almost completely inhibited parasite growth *in vitro*. This trial, the first chimeric protein based malaria vaccine candidate to be tested in human trials demonstrates favorable antibody levels to the vaccine antigen in comparison to that in other phase I clinical trials with other malaria vaccine candidates [23], [24], [29].

Consistent with observations in other clinical trials using the Montanide ISA720 adjuvant, our vaccine formulated with this adjuvant caused a high frequency of local adverse events. Although difficulties in differentiating induration and nodules led to the classification of suspected nodules as induration in this trial, it was clearly observed that some of the events such as swelling, induration and suspected nodules were related to the higher doses and increasing number of injections. In addition, since the conduct of this trial, various international efforts to improve the detection of vaccine safety data, such as the Brighton Collaboration case definition and guidelines for nodule, swelling and induration have been published and in future clinical testing of this formulation [30], [31], [32], these definitions will be used to guide of the detection and diagnosis of adverse events.

In conclusion, the data from this study as well as our previous investigations [22], [33], [34] support the PfCP-2.9/ISA720 as a promising blood-stage malaria vaccine for further clinical development. The vaccine formulation was immunogenic, and all of the vaccinated participants seroconverted to the antigen. It was encouraging that the vaccine induced antigen specific antibodies, and that the antibodies recognized native proteins on the parasite. Efforts to further improve the formulation and optimize dose and schedule of vaccination are being continued in a second clinical trial evaluating lower vaccine doses, and a variation in the intervals between vaccination [35]. It is hoped that this will lead to achieving the appropriate balance between vaccine immunogenicity and reactogenicity in future clinical development of this promising malaria vaccine candidate.

## Supporting Information

Data S1 (0.31 MB DOC)

Data S2 (0.12 MB DOC)

Protocol S1 Protocol of WHO-sponsored trial
(2.10 MB DOC)

Checklist S1 CONSORT checklist of PfCP-2.9
(0.06 MB DOC)
