# Supplementary material for: Safety and Immunogenicity of a Malaria Vaccine, Plasmodium falciparum AMA-1/MSP-1 Chimeric Protein Formulated in Montanide ISA 720 in Healthy Adults
Source: PLoS One. 2008 Apr 9;3(4):e1952. doi: 10.1371/journal.pone.0001952 (PMC2276862; doi:10.1371/journal.pone.0001952)
Supplement: Data S1 — (0.31 MB DOC) [file pone.0001952.s001.doc]

## Supplementary data 1: Manufacture and characterization of PfCP-2.9

## recombinant protein

1. Fermentation:

The conditions for the fermentation of the PfCP-2.9 expressing strain *P. pastoris* (3N25) were optimised to achieve high level production of the protein. These include methanol-induction concentration, range of pH value, timing of induction, cell density and optimal dissolved oxygen levels, etc. 500ml culture of yeast grown at 30 ^o^C for 22 hrs was inoculated into a 30-litre fermentor containing 12 litre of minimal salts fermentation medium . Figure 1 showed a SDS-PAGE analysis of samples from the fermentation at various time points. As shown in this figure, the concentration of the recombinant protein in the fermentor supernatant increased during the period of fermentation and the yield of the product was higher than 1g/liter.

2. Purification:

The protein was produced at the 30-L scale in Wanxing Bio-pharmaceutical company. The protein was purified by using three steps including hydrophobic interaction, ion-exchange and gel filtration chromatography. The percentage of protein recovered from each step is indicated in Table 1.

0 4 12 16 20 24 28 32 36 44 48 52 56 62 70 74 80 88 92 96hrs

**
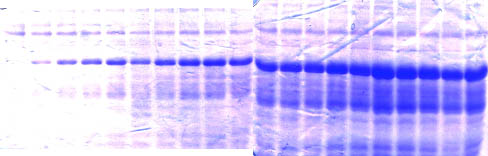
**

**Fig.1 SDS-PAGE analysis of time-point samples of the fermentation**

8 μl of the fermentation supernatant at various time points after induction of the expression by methanol was loaded on each lane of the SDS-PAGE gel. The product is indicated by an arrow.

**Table 1, Purification of the PfCP-2.9 recombinant protein**

Purification steps

volume (ml)

Product yield

(mg/ml)

Step rate（%）

Total rate

（%）

microfiltration

**4800**

**1.261**

**100**

**-**

phenyl

**2490**

**1.78**

**73.2**

**73.2**

DEAE

**395**

**7.15**

**63.7**

**46.6**

Superdex 75

**1140**

**2.29**

**92.4**

**43.1**

3. Purity of the product

The recombinant protein was purified to near homogeneity from the yeast supernatant as shown in Fig.2 and Fig. 3. The purity of the product was analysed by both SDS-PAGE and HPLC as shown in table 2.

4. Interaction of the Protein with monoclonal antibodies.

Since MSP1-19 and AMA-1 are cysteine-rich proteins and as their functional antibodies are disulfide bond-dependent, it is extremely important to retain all conformational epitopes after fusion of the two proteins into single molecule. To gain an insight into conformational properties of the chimeric protein, we analysed the interaction of PfCP-2.9 with 6 specific monoclonal antibodies including two *Plasmodium falciparum* inhibitory antibodies. All the antibodies recoganize conformational epitopes. The results show that all these antibodies interact with PfCP-2.9 in a reduction-sensitive manner, indicating that at least the corresponding six important epitopes of the chimeric protein resemble identical to native ones.


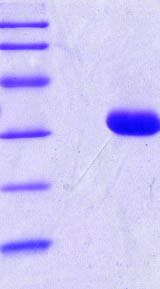


Fig. 2 SDS-PAGE analysis of purified PfCP-2.9 recombinant protein


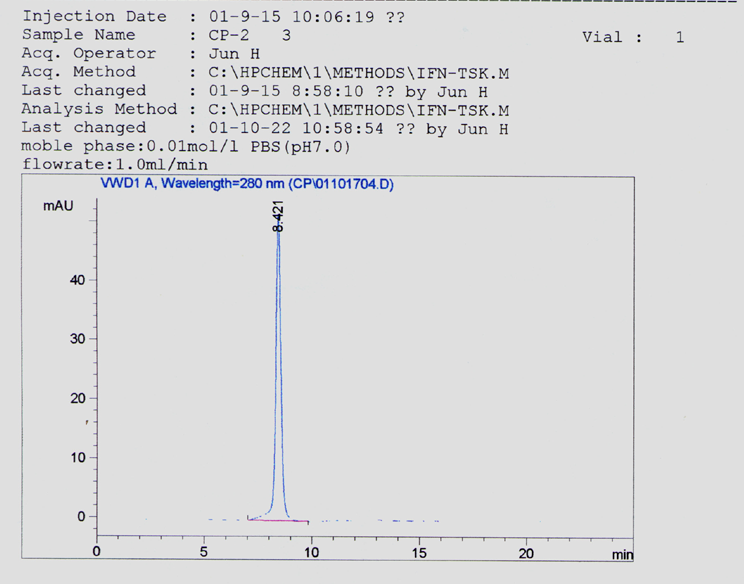


Fig. 3 HPLC analysis of purified PfCP-2.9 recombinant protein

5. Other characterizations of the recombinant protein( see Table 2)

**Table 2 Summary of QA/QC results for PfCP-2.9 protein**

Test results

Concentration(mg/ml) 2.29

Purity SDS-PAGE(non-reduced) 100%

HPLC 99.3%

MW 34,608

Host DNA content(pg/dose) <100

PI 4.7

Host protein content 0.1%

Peptide mapping identical*

N-terminal sequence ALSHPIEVENNFPCS

Endotoxin level(EU/dose)** <2.5

Microbial content (CFU/ml) <0

* identical from batch to batch.

** Entotoxin content of the purified protein (prior to formulation) was measured by the Limulus Amebocyte Lysate (LAL) assay.
