# Supplementary material for: Safety and Immunogenicity of a Malaria Vaccine, Plasmodium falciparum AMA-1/MSP-1 Chimeric Protein Formulated in Montanide ISA 720 in Healthy Adults
Source: PLoS One. 2008 Apr 9;3(4):e1952. doi: 10.1371/journal.pone.0001952 (PMC2276862; doi:10.1371/journal.pone.0001952)
Supplement: Data S2 — (0.12 MB DOC) [file pone.0001952.s002.doc]

**Supplementary data 2:**

**Stability and potency of PfCP-2.9 formulation lots used in the clinical trial**

Based on stability and potency of the pre-clinical vaccine formulation lots, we developed a protocol for manufacturing the vaccine emulsion that was confirmed to be stable in physical and biological properties. We used this protocol to produce four lots of the vaccine formulation for the clinical study including lots of 400 µg, 200 µg, 100 µg and 40 µg . Physical and biochemical stability and potency of the vaccine lots were evaluated for six months.

For measurement of protein stability, the vaccine emulsion was packaged into autoclaved 2 ml bottle with 1 ml volume of emulsion and stored at 4℃ for various periods. Extract of the protein from the emulsion was performed by the Seppic’s Corporation Instruction. Briefly, 200µl of benzyl alcohol was added to 1.0 ml the formulation. After vortexed for 5 minutes, the mixture were transferred to the microcentrifuge tube and then centrifuged at 2500g for 20 minutes. The middle aqueous layer was carefully obtained from the three-phase system. The extracted protein were electrophoresed on 10% polyacrylamide SDS gels and stained by commas-blue to determine any degradation of the antigen. As showed in figure 1, four lots of the formulation were stable during the period of six month. No degraded band of this protein was detected by SDS-PAGE with Commas-blue staining.

A B C

1 2 3 4 M

1 2 3 4 M

1 2 3 4 M


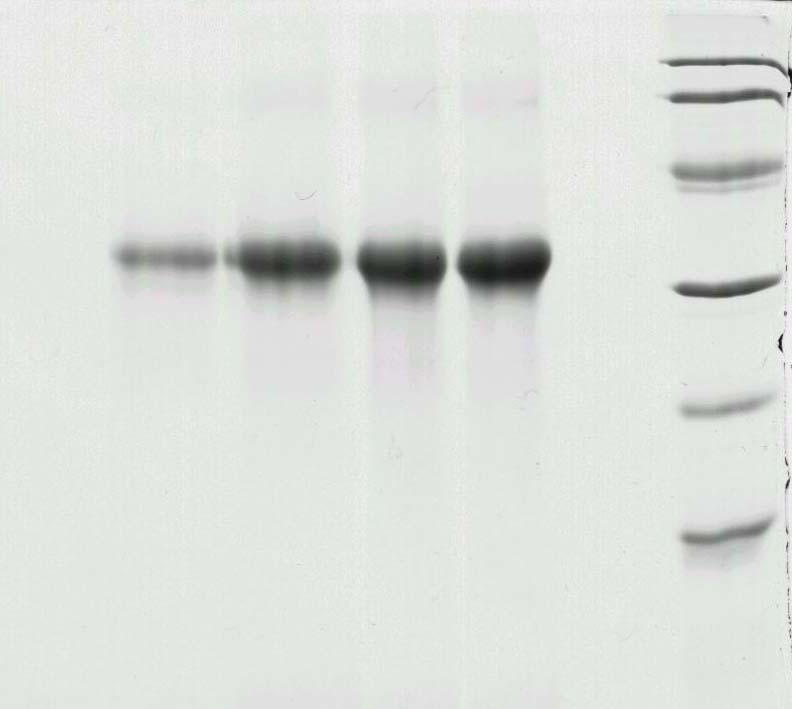


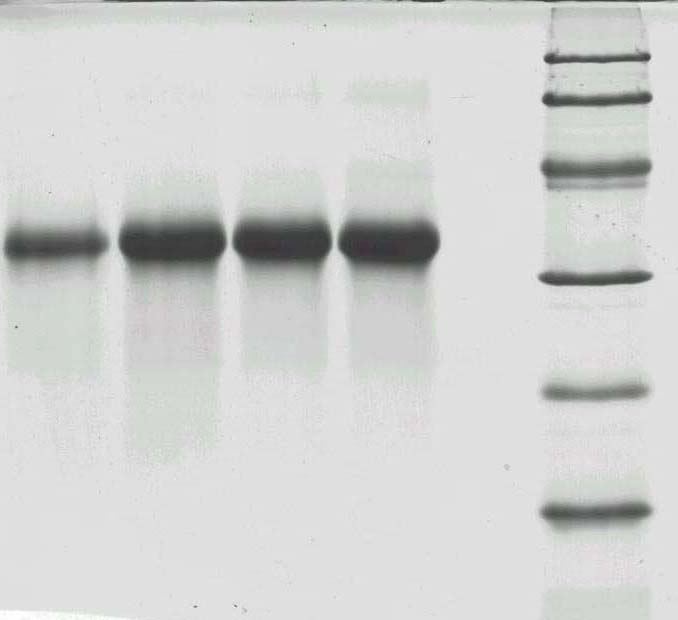


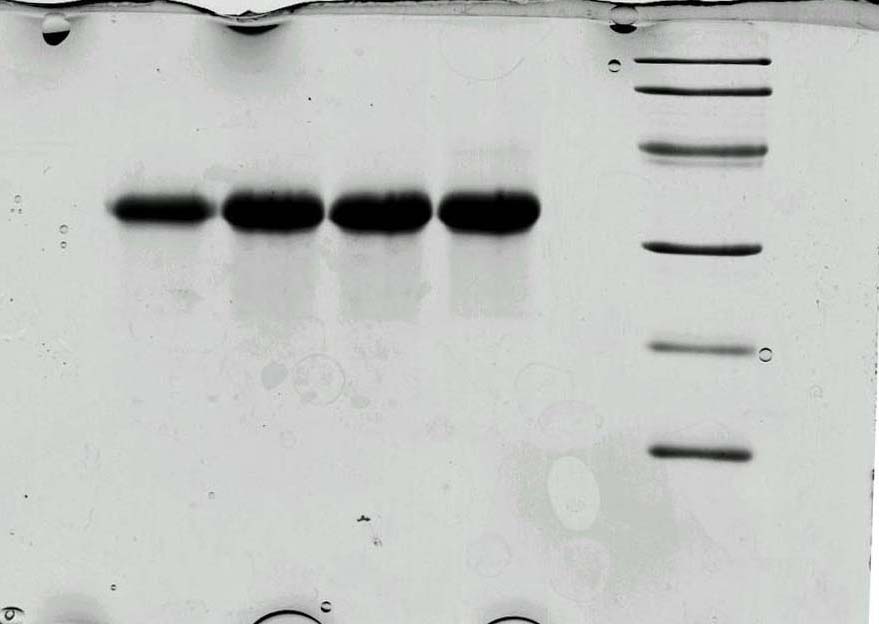


Figure 1 Detection of the PfCP-2.9 protein extracted from vaccine emulsion by SDS-PAGE with Commas-blue staining.

A: 0 month; B: 3 months; C: 6 months;

Lane 1~4: lots of 40 µg , 100 µg , 200 µg and 400 µg, respectively

Immunological potency test of the vaccine formulation was carried out in six groups of BALB/c mice( ten per group). The animals were intraperitoneally injected at a single dose of 0, 0.01, 0.04, 0.16, 0.64, and 2.56 µg of PfCP-2.9 formulation either fresh or after storage for 3 and 6 months at 4 ℃. Blood was collected four weeks after immunization and the specific antibodies of serum samples were detected by ELISA. According to positive ratio of sercoversion each group, ED_50_ was calculated for each vaccine formulation. The results from this assay indicated that ED_50_ of the vaccine emulsion stored for 0, 3 and 6 months at 4℃ were 0.037, 0.032 and 0.033µg, respectively, which showed no significant changes between the fresh formulation(0 month) and post-storage formulation for 3 and 6 months.

Table 2 Positive seroconversion rate of specific antibody and its ED_50_ in different groups immunized with various doses of PfCP-2.9 vaccine from 200μg/ml vaccine formulations stored for 0, 3 and 6 months, respectively.

| period of storage | Seroconversion rate  Dose（μg） | | | | | | ED_50_ (μg) |
| --- | --- | --- | --- | --- | --- | --- | --- |
|  | 0 | 0.01 | 0.04 | 0.16 | 0.64 | 2.56 |  |
| 0 month | 0/10 | 1/10 | 5/10 | 10/10 | 9/10 | 10/10 | 0.037 |
| 3 months | 0/10 | 1/10 | 8/10 | 10/10 | 10/10 | 10/10 | 0.032 |
| 6 months | 0/10 | 1/10 | 7/10 | 8/10 | 10/10 | 10/10 | 0.033 |
