# Supplementary material for: Safety and Immunogenicity of a Malaria Vaccine, Plasmodium falciparum AMA-1/MSP-1 Chimeric Protein Formulated in Montanide ISA 720 in Healthy Adults
Source: PLoS One. 2008 Apr 9;3(4):e1952. doi: 10.1371/journal.pone.0001952 (PMC2276862; doi:10.1371/journal.pone.0001952)
Supplement: Protocol S1 — Protocol of WHO-sponsored trial (2.10 MB DOC) [file pone.0001952.s003.doc]

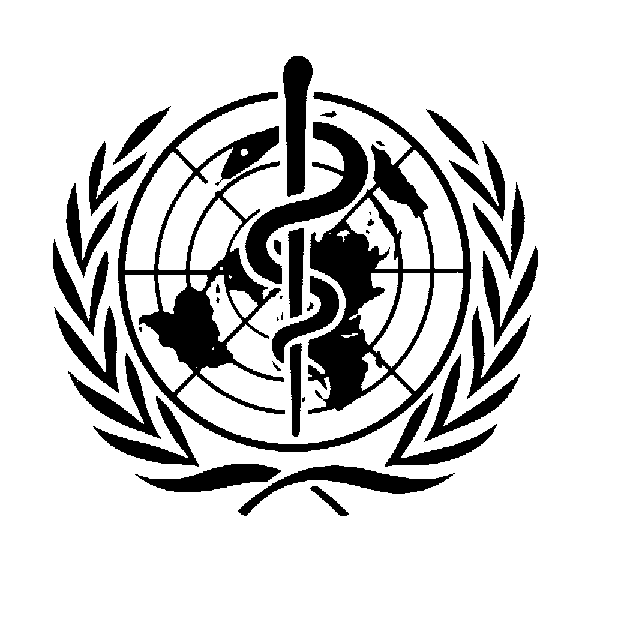


**WORLD HEALTH ORGANIZATION**

**CONFIDENTIAL PROTOCOL OUTLINE**

**Project Title**

Blood-Stage PfCP-2.9 Recombinant Vaccine against *Plasmodium falciparum*

**Protocol title**

**A Phase 1 randomized singled blinded single center study, comparing doses of PfCP-2.9 recombinant vaccine adjuvanted with Montanide ISA 720 for safety and immunogenicity**

**STUDY PRODUCTS**

***Plasmodium falciparum* Chimeric Protein 2.9 (PfCP-2.9) adjuvanted with Montanide ISA 720**

**Project ID No. / Study ID: S001**

**Protocol version date: Draft No. 08 dated on 28 March 2003**

**Protocol approval by:**

**Clinical Coordinator ______________Date__/__/2003**

**WHO Internal** **Project Manager ______________Date__/__/2003**

| **Principal Investigator:** | |
| --- | --- |
| *Dr.Jinhong Hu*  *Clinical Center of Pharmacology*  *Changhai Hospital, Second Military Medical University*  *174 Changhai Rd., Shanghai 200433, China*  *Tel: +86-021-25070665*  *Fax: +86-021-25070665* | |
| **WHO Clinical Monitor** | Dr Fe Esperanza Espino |
|  | Dr.Yupaporn Wattanagoon |
| **WHO Clinical Coordinator** | Dr. Juntra Karbwang, |
| **WHO Internal Project Manager** | Dr. Deborah Kioy |
| **Wanxing Clinical Monitor** | Mr.Fei Xie, Dr. Haitao Liu |

I, the undersigned, have reviewed this protocol, including attached information and I will conduct the clinical study as described and will adhere to the Ethical and Regulatory Considerations.

| **Investigator Signature:** |
| --- |
| **Date:** 28 / 03 / 2003 |

| PROTOCOL TITLE | A Phase I randomized single blinded single center study, comparing doses of PfCP-2.9 recombinant vaccine adjuvanted with Montanide ISA 720 for safety and immunogenicity |
| --- | --- |
| LICENSED NUMBER | 2002SL0046 |
| SPONSOR | WHO/TDR |
| PROJECT PHASE | Phase I |
| INDICATION UNDER STUDY | Plasmodium falciparum malaria |
| OBJECTIVE (S) | Primary: To assess the safety and reactogenicity of PfCP-2.9 vaccine in healthy adult volunteers.  Secondary: To assess the immunogenicity of PfCP-2.9 vaccine in healthy adult volunteers |
| EXPERIMENTAL DESIGN  OF THE TRIAL | Single-blind, increasing dose (staged), randomized within group, single center |
| PLANNED SAMPLE SIZE | 52 enrolled, 40 verum, 12 placebo available for analysis |
| NUMBER OF CENTERS | Single center |
| SUBJECT SELECTION  CRITERIA | Healthy Chinese male and female adults (18 to 45 years old) |
| NAME AND FORMULATION OF DRUG | Injectable emulsion containing 40μg/ml, 100μg/ml, 200μg/ml or 400 ug/ml of PfCP-2.9 recombinant protein, formulated with Montanide ISA 720 |
| DOSAGE AND SCHEDULE | 20, 50, 100 and 200 μg of protein per dose (0.5 ml) per volunteer in 1 dose. Dose to be repeated at same dosage on Day 60 and 180. |
| ROUTE OF ADMINISTRATION | i.m. In left lateral deltoid (first injection); in right lateral deltoid (second injection) and in left lateral deltoid (third injection). |
| COMPARATOR | Montanide ISA 720 adjuvant |
| **PARAMETERS OF** |  |
| **SAFETY:** | 1. Local and systemic tolerability  2. Reported adverse events (AES)  3. Clinical and laboratory values |
| **IMMUNOGENICITY:** | 1. Antibody titres by ELISA 2. Antibody titers by IFAT   3. Lymphocyte stimulation indices  4. Antibody function by GIA |

**Summary of Clinical Protocol:**

**A *Plasmodium falciparum* Chimeric Protein 2.9 (PfCP-2.9) has been produced under GMP conditions in *Pichia Pastoris* consisting of two promising malaria vaccine candidates, AMA-1 (III) and MSP1-19. PfCP-2.9 has been formulated with Montanide ISA720 adjuvant. In this initial phase I dose-escalation study, the primary objective will be to evaluate the safety and reactogenicity of the vaccine candidate in healthy adults in Shanghai, China. In addition, the immunogenicity of the vaccine candidate will be assessed as the secondary objective and the optimal dose for human use will be identified. For these objectives, 52 volunteers whose conditions match all the inclusion and none of the exclusion criteria will be randomly allocated to five groups, 40 verum and 12 comparators. Injections will be administered according to a 0, 60, 180 day schedule. Vaccination will be staggered starting with the 20 ug cohort. The procedure for the vaccination is following: On day 0, 3 verum in group 1 and 1 comparator in group 5 will be injected i.m. in the left lateral deltoid with 0.5 ml of the vaccine emulsion containing 20 μg of the antigen for group 1 and no antigen for group 5. After two-week’s review of adverse events (AEs) associated with the vaccination above, if no severe or serious AEs, the remaining 7 of group 1 and 2 of group 5 will be immunized. This vaccination procedure will be followed for the higher doses in groups 2, 3 and 4. All the safety data in a group will be reviewed by the DSMB prior to proceeding to the next higher dose. Volunteers will be interviewed on Day 0, 1, 2, 7, 14, 30, 60, 61, 62, 74, 90, 180, 181, 182, 194 and 240. Blood samples will be taken at screening, on Day 0, 30, 60, 90, 180, 194 and 240 for evaluation of clinical chemistry, hematology and immunological analyses including humoral and cellular responses. Adverse events assessment will be separated into local and systemic AEs and the intensity of each AE will be graded to 3 levels. All immunological analyses will be performed in agreement with SOPs available on site. All trial data will be recorded on the Case Report Form.**

| **TABLE OF CONTENTS** | **Page No** |
| --- | --- |

1 General Information 7

1.1 Protocol title 7

1.2 Sponsor 7

1.3 Authorized person(s) to sign the protocol and the protocol amendment(s) 7

1.4 Sponsor’s medical expert for the trial 7

1.5 Principal (PI) and Sub-Investigator 7

1.6 Trial Site Address 8

1.7 Clinical Laboratories 8

1.8 Study Consultants 8

1.9 Vaccine 9

1.10 Data management team 9

1.11 DSMB (Data Safety Monitoring Board) 9

2 Background Information 10

3 INVESTIGATIONAL PRODUCT INFORMATION………………………….......11

4 Trial Objectives and Purpose 12

4.1 Primary objectives 13

4.2 Secondary objectives 13

5 Trial Design 13

5.1 Trial endpoints 13

5.2 A description of the type/design of trial to be conducted 13

5.3 DESCRIPTION OF THE MEASURES TAKEN TO MINIMIZE/AVOID BIAS:…………………………………………………….16

5.4 DESCRIPTION OF INVESTIGATIONAL PRODUCT ………………………………………………………… ……………………… 16

5.5 Description of the sequence and duration of the trial periods including follow-up

and expected duration of volunteer participation 16

5.6 Identification numbers 17

5.7 Description of "discontinuation criteria" for individual volunteers, and for the trial 17

5.8 Minimum information to be recorded in the source documents 18

5.9 Identification of data to be recorded directly on the Case Report Form (CRFs) 18

6 Inclusion and exclusion criteria for vaccination 18

6.1 Volunteer inclusion criteria for vaccination 18

6.2 Volunteer exclusion criteria for vaccination 19

6.3 Vaccination 19

6.4 Other medication(s)/treatment(s) authorized (including rescue medication) and not

authorized before and/or during the trial. 20

7 Trial procedure 20

7.1 Recruitment and screening 20

7.2 Day 0 / Vaccination 21

7.3 Follow-up visits 25

7.4 Procedures for monitoring volunteer compliance. 26

7.5 Contraception methodse. 27

8 Assessment of Safety 27

8.1 Materials and Methods 27

8.2 ADERSE EVENT(AES) REPORTING………………………………………………………………...……………….30

8.3 Criteria for safety 31

9 Assessment of immunogenicity 31

9.1 Materials and Method for the assessment of the immunogenicity endpoints 31

9.2 Criteria for immunogenicity 31

10 Statistical analysis 32

10.1 Sample size calculation 33

10.2 Safety analysis 33

10.3 Immunogenicity analysis 33

10.4 Procedure for accounting for missing, unused, and spurious data. 33

10.5 Procedures for reporting any deviation(s) from the original statistical plan 34

11 Direct access to source data/documents 34

12 Quality control and quality insurance 34

12.1 Essential documents for trial initiation 34

12.2 Archiving 35

13 Data handling and management 35

14 Ethical Aspects 36

14.1 Approval by the local IRB/IEC and SCRIHS 36

14.2 Volunteer Information and Informed Consent 36

15 Financing and insurance 37

16 Publication policy 37

17 Protocol amendment 37

18 Consent Form 37

19 Abbreviations 38

20 References 38

APPENDIX 1: Common Toxicity Criteria

APPENDIX 2: Procedures for the measurement of immunological markers

APPENDIX 3: insurance statement from insurance company

**APPENDIX 4: Assessment table for Phase I Clincal Trial for DSMB**

# 1. General Information

## Protocol title

A Phase 1 randomized single blinded single center study, comparing doses of PfCP-2.9 recombinant vaccine adjuvanted with Montanide ISA 720 for safety and immunogenicity

## Sponsor

World Health Organization, Special Programme for Research and Training in Tropical Diseases (TDR), 20 Av. Appia, CH-1211, Geneva-27 Switzerland

## Authorized person(s) to sign the protocol and the protocol amendment(s)

**Clinical Co-ordinator** Dr Juntra Karbwang,

WHO, TDR, PRD,

20 Avenue Appia, CH-1211, Geneva 27, Switzerland

Phone: 41-22-791-3867, Fax: 41-22-791-4854

E-mail: [karbwangj@who.int](mailto:karbwangj@who.int)

**Internal Project Manager** Dr Deborah Kioy

WHO, TDR, PRD,

20 Avenue Appia, CH-1211, Geneva 27, Switzerland

Phone: +41-22-791-3591, Fax: +41-22-791-4825

E-mail:

## Sponsor’s medical expert for the trial

Dr Juntra Karbwang,

WHO, TDR, PRD,

20 Avenue Appia, CH-1211, Geneva 27, Switzerland

Phone: 41-22-791-3867, Fax: 41-22-791-4854

E-mail: [karbwangj@who.int](mailto:karbwangj@who.int)

## Principal (PI) and Sub-Investigators

Principal Investigator:

*Dr. Jinhong Hu*

*Clinical Center of Pharmacology*

*Changhai Hospital, Second Military Medical University*

*174 Changhai Rd, Shanghai 200433, China*

*Tel: +86-021-25070665*

*Fax: +86-021-25070665*

Sub-investigators:

*Dr. Mobin Wan*

*Department of infection disease*

*Changhai Hospital, Second Military Medical University*

*174 Changhai Rd, Shanghai 200433, China*

*Tel: +86-021-25070609*

*Fax: +86-021-25070609*

*Dr. Zhihui Chen*

*Department of infection disease*

*Changhai Hospital, Second Military Medical University*

*174 Changhai Rd, Shanghai 200433, China*

*Tel: +86-021-25070613*

*Fax: +86-021-25070609*

*Dr.Jun Gu*

*Department of dermatology*

*Changhai Hospital, Second Military Medical University*

*174 Changhai Rd, Shanghai 200433, China*

*Tel: +86-021-25070622*

*Fax: +86-021-25070622*

## Trial Site Address

Changhai Hospital, 174 Changhai Rd, Shanghai 200433, China

## Clinical Laboratories

Dr. Qian Shen

Department of Laboratory Diagnosis

Changhai Hospital, Second Military Medical University

174 Changhai Rd, Shanghai 200433, China

Tel: +86-021-25070648

Fax: +86-021-25070648

Dr. Wenjie Li

Department of Laboratory Diagnosis

Changhai Hospital, Second Military Medical University

174 Changhai Rd, Shanghai 200433, China

Tel: +86-021-25070641

Fax: +86-021-25070641

## Study Consultants

Dr. Blasie Genton

Swiss Tropical Institute

Socinstrasse 57

4002 Basel, Switzerland

Tel: 41-61-2848 130

Fax: 41-61-2718 654

Email: Blaise.Genton@hospvd.ch

Dr Marie-Paule Kieny

Director, Initiative for Vaccine Research

World Health Organization

Avenue Appia 20, CH1211-Genève 27

Tel: +41 22 791 35 91

Fax: +41 22 791 48 60

E-mail: [kienym@who.int](mailto:kienym@who.int)

## Vaccine

*Plasmodium falciparum* Chimeric Protein 2.9 (PfCP-2.9) adjuvanted with Montanide ISA720

Injectable emulsion containing: 40μg/ml, 100μg/ml, 200μg/ml /ml and 400μg/ml /ml of PfCP-2.9 recombinant protein, prepared by formulation with Montanide ISA 720 to give 20, 50, 100 and 200μg of protein per dose (0.5 ml)

## Data management team

Drs. Jia He

Department of Statistics, Second Military Medical University,

800 Xiang Yin Rd., Shanghai 200433, China.

Tel: +86-021-25070418

Fax: +86-021-25070418

## DSMB (Data Safety Monitoring Board)

### Members

### Dr. Vichai Chokevivat (Chair of DSMB)

### Department for Development of Thai Traditional and Alternative Medicine

### Ministry of Public Health

### Nonthaburi, Thailand 11000

### Phone: +66 1 811 7586

### E-mail: vichai@health.moph.go.th

### Dr. Marilla Lucero

### Research Institute for Tropical Medicine

### Filinvest Corporate City

### Alabang, Muntinlupa

### Manila, Philippines.

### Phone: 639178918531

### E- mail: mglucero@pacific.net.ph

Dr. Jianmin Wang

Department of hematology

Changhai Hospital, Second Military Medical University

174 Changhai Rd, Shanghai 200433, China

Tel: +86-021-25074610

Fax: +86-021-25074610

### Functions

- - Evaluates safety data following review of 14 days of safety data (local and systemic assessment) for each dose.
  - Makes recommendations to the PDT (Product Development Team) for continuing of clinical development of PfCP-2.9 malaria vaccine.
  - Can suspend the trial for reasons of safety.
  - Interacts by electronic communication or through meetings, as requested.

# 2. Background Information

Malaria is one of the most important causes of ill health in many tropical and subtropical regions of the world. The annual incidence of clinical disease caused by malaria parasites is estimated to be 300-500 million and approximately 40% of the world’s population live in areas where there is some risk of infection. Of the four species of human malaria parasite, *Plasmodium falciparum* is most virulent killing an estimated 1 to 2 million children annually, mostly in sub-Saharan Africa. The emergence of drug-resistant parasites and *Anopheles* mosquitoes that are resistant to insecticides have contributed to the persistence and in many cases worsening of the malaria problem. For these reasons the development of a vaccine against malaria is a major global public health priority.

Vaccination is a tool used to control several diseases including eradication of smallpox from the world and polio from the Western Hemisphere. Although this type of sterile protective immunity does not exist from naturally acquired malaria, there are several reasons for believing that malaria vaccine is feasible: (1). In malaria-endemic regions, parasite density levels decrease with age and the clinical manifestations of malaria are generally much milder in adults and older children than in infants and children under 5 years of age. This indicates that repeated infection induces immune responses that reduce parasite burden and the morbidity and mortality of malaria; (2). Immunization of volunteers with radiation-attenuated sporozoites confers sterile protective immunity; (3). Protective immunity has been induced in various animal model of malaria by immunization of animals with native as well as recombinant proteins; and (4). Immunoglobulin purified from the blood of adults who lived their entire lives in malaria-endemic regions can passively transfer protection against *P. falciparum*, indicating that antibodies against antigen of blood-stage can have an effect on eliminating the parasite.

Based on the life cycle of the malaria parasite, there are several vaccine targets including the pre-erythrocytic stage (sporozoite, hepatic stages), the erythrocytic stage and the sexual forms of the parasite. For the pre-erythrocytic stage, various clinical trials with the circumsporozoite protein indicated some efficacy, and much effort has been directed into improving the immunogenicity of the vaccine. Since only the asexual erythrocytic stage is associated with pathology and the disease, an effective vaccine against this stage will prevent the development of clinical malaria and the risk of death. The prospects for an effective blood-stage vaccine have been brightened by the realisation that there are several blood-stage antigens capable of inducing immune responses that can inhibit the growth or development of the parasite.

In the last decades, several malaria vaccine candidates have been evaluated in human including SPf66 ^[1-3]^, a vaccine that has been tested extensively in clinical trials. Although the vaccine provided some efficacy in trials in Colombia and Tanzania, in later trials it was shown to give no protection and consequently, SPf66 is unlikely to become a widely used vaccine against malaria. RTS, S is another vaccine candidate based on the circumsporozoite protein which was shown to be promising candidate in recent trials, but the duration of the immunity was shown to be short ^[4,5]^. During the past 20 years enormous progress toward achieving an effective vaccine has been made by numerous scientists throughout the world, but a safe, highly effective malaria vaccine is still not available.

1. **The investigational products information**

**Rationale for PfCP-2.9 vaccine**

The vaccine to be used in this study contains recombinant PfCP-2.9 protein consisting of two components, AMA-1 (III) and MSP1-19 of *Plasmodium falciparum*. The ~200kDa Merozoite Surface Protein-1 (MSP1)^[6-17]^ and the Apical Membrane Antigen (AMA-1) ^[18,19]^ of *Plasmodium falciparum* are attractive malaria vaccine antigens. These two antigens are located on the parasite surface and are believed to play a role in the invasion process. The most convincing evidence for the inclusion of MSP1 comes from monkey experiments where immunization of Aotus monkey provides partial to complete protection against a challenge of *Plasmodium falciparum*. The portion of MSP1 targeted by protective immunity has been mapped to the 19 kDa carboxy-terminal region (MSP1-19), which contains two epidermal growth factor (EGF) like domains. Monoclonal antibodies as well as rabbit antibodies against this region inhibit parasite growth in vitro. Immunization with a recombinant protein corresponding to this region in mouse model can induce protective immunity against challenge.

AMA-1 is a protein found in the apical organelles of malaria merozoites. It is discharged onto the merozoite surface during or prior to invasion. AMA-1 has been characterized from human, monkey and rodent parasites. There is considerable evidence that AMA-1 is the target of protective immune responses:

- AMA-1 is a target of antibodies that inhibit growth in vitro;
- *P. Chabaudi* AMA-1 induces active immunity which protects mice against homologous challenge and has even demonstrated sterile immunity;
- *P. Fragile* AMA-1 protected *Saimiri* monkeys against challenge with P. fragile. Parasites grew more slowly in immunized animals, and this decrease in initial growth rate correlated with antibody levels;
- *P.chalbaudi* AMA-1 induces antibodies in rabbits, which protect mice against challenge with *P. chabaudi* after passive immunization;
- *P. Falciparum* AMA-1 induces antibodies in rabbits which inhibit the development of *P. falciparum in vitro*;

A three-domain sub-structure to the AMA-1 ectodomain was recently described and the most C-terminal of the disulphide-bonded domains in AMA-1 (Domain III), which has a "cysteine knot-like" structure, may also be carried in on the surface of the invading merozoite as occurs with MSP1-19.

Because both MSP1 and AMA-1 are promising malaria vaccine candidates, we have constructed a *Plasmodium falciparum* Chimeric Protein 2 (PfCP-2.9) comprising the AMA-1 (III) from 3D7 line and MSP1-19 from k1 line of *Plasmodium falciparum* respectively. The two individual proteins were fused via a hinge encoding a Gly-Pro-Gly-Pro motif repeat as well as a small peptide encoded by a multiple cloning site (mcs) to generate PfCP-2.9 protein. The amino acid sequence of PfCP-2.9 was translated into a DNA sequence in which the codon usage was designed to optimize expression of the gene in yeast. Three potential glycosylation sites on this chimeric protein were eliminated by changing codons from Asn to Gln. The resulting PfCP-2.9 is a 26.86 kDa of chimeric protein consisting of 241 amino acid residues and contains eighteen cysteine residues, of which six are located in AMA-1 (III) and the rest in MSP1-19, to form nine intramolecular disulfide bonds. To synthesize the gene, the sequence of the gene was divided into eight oligonucleotide primers of about 80 nt in length. The overlapping region between two primers varied from 15 to 20 nt long. The eight primers were assembled by using Asymmetric PCR based Method we developed to generate 723 bp of PfCP-2.9 gene. The resulting gene was expressed and secreted at high levels in shaker flasks with a yield of 815 mg/litre whereas of 2600 mg/liter in 15-L scale fermentation. More than 40% of the protein was recovered from a three-step purification process with >98% purity.

The purified protein solution was formulated with adjuvant Montanide ISA 720 by mixing 70% of the adjuvant with 30% of the antigen solution using a Homogeneizer at 4000 rpm for 4 min. The quality of the emulsion was controlled by several parameters including droplet test, conductivity, particle size and stability. After examination for quality, the emulsion was packaged into autoclaved 2ml vials with 1ml volume of emulsion, which contains various concentration of the vaccine antigen.

All these operations were conducted in accordance with GMP.

**Background on Vaccines Formulated with Montanide ISA 720**

Montanide ISA 720 has been used as an adjuvant in nine clinical trials of vaccines delivered intramuscularly ^[20-28]^. Within the nine clinical trials, 426 doses of Montanide ISA 720 containing vaccine were given to 192 human volunteers. The antigens were all recombinant proteins or synthetic peptides and were delivered in two or three dose regimens, at concentrations ranging from 5ug to 1000ug, with the time between injections ranging from 4-26 weeks. Adverse events observed during those trials are summarized below:

Within these trials, 210 individual doses of Montanide ISA 720 without any antigen were given to 104 human volunteers. Montanide ISA 720 was given in 1, 2 or 3 dose regimes, with volumes ranging from 0.36 ml to 1.8 ml, with 4 to 24 weeks between doses. There were no serious or severe adverse events reported.

The initial set of clinical trials led to a concern about acute and delayed local reactogenicity associated with vaccines that contain Montanide ISA 720. The acute reactions consisted of pain, swelling, induration, sterile abscess, and granuloma formation. The overall rate for mild and moderate acute local reactions was 54%, which compares favorable with the rate of local reactions seen with pediatric vaccines such as DT (55% mild/moderate local reactions) or dT (52% mild/moderate reactions) in 6-year-old children. Local reactions, which were graded as severe, occurred only with three vaccines for a cumulative rate of 4%. This also compares favorably to the licensed vaccines (17% severe local reactions with DT and 11% severe local reactions with dT).

Two vaccine formulations ^[22,24,26]^ led to the development of delayed local reactions seen 10-14 days after the vaccine was injected. A total of 17 individuals from a total of 426 doses (crude rate of 4%) developed pain and swelling at the injection site in this delayed time frame. All signs and symptoms completely resolved within days to weeks. Only three individuals had severe delayed reactions and these occurred in individuals that received at least 150ug of antigen.

Overall, both the local and delayed reactions appear to be antigen specific and are not predicted using animal models. Furthermore, there appears to be a trend for increased rate and severity of local reactions with increased antigen concentration.

# 4. Trial Objectives and Purpose

The objective of the study is to evaluate the safety, optimal dose and immunogenicity of PfCP-2.9 blood-stage malaria vaccine. The study will include a Comparator group, Montanide ISA720 adjuvant.

## 4.1 Primary objectives

### Evaluate the safety and reactogenicity of PfCP2.9 vaccine in healthy adult volunteers.

### 4.2 Secondary objectives*.*

### - Assess the immunogenicity of PfCP2.9 in healthy adult volunteers

### - Identify the dose(s) of PfCP2.9 vaccine giving optimal human immune response.

**5. Trial Design**

## 5.1 Trial endpoints

### Primary endpoints

#### Occurrence of local and/or systemic adverse events (AEs) after each vaccination assessed by the tabulation of unsolicited and solicited adverse events including interview, physical examination, hematology and clinical chemistry.

### Secondary endpoints

#### Antibody response after each vaccination assessed by ELISA as well as IFA.

#### Cellular response after vaccination assessed by lymphocyte proliferation assay with PfCP-2.9 recombinant vaccine.

#### Assessment of antibody function via inhibition of parasite growth *in vitro* by immune sera

## 5.2 Type/design of trial to be conducted

This is a single blind, randomized, escalating dose, and placebo controlled single centre study. The study is the initial Phase I study of this vaccine candidate. The group sizes were selected to allow for the initial assessment of vaccine safety and tolerability without exposing a larger number of volunteers to an experimental vaccine.

During weeks -2 to 0 volunteers will be screened and allocated to one of five groups (10 or 12/group) with the aid of a table of random numbers. Within these groups, volunteers will be randomized into verum (4 groups) and placebo (1 group) recipients. On day 0, 60 and 180 they will be injected i.m. in the deltoid with PfCP-2.9 in Montanide ISA720 adjuvant in a volume of 0.5ml. After each injection volunteers will be observed for 24 hour to identify and treat immediate adverse events. Instruction on use of diary cards will be given to volunteers to record solicited adverse events daily for 14 days post injection. After the initial injection each volunteer will be seen at 24, 48 hours, day 7 and day 14. The injection site will be examined and the subjects asked about adverse events. The diary cards will be collected at day 14 visits. In the same way subjects will be seen and examined according to the same schedule for all 3 vaccine doses.

Volunteers will be interviewed on Day 0, 1, 2, 7, 14, 30, 60, 61, 62, 74, 90, 180, 181, 182, 194 and 240. Blood samples (10-20 ml) will be taken at screening and on Day 0, 30, 60, 90, 180 and 240. On Day 194, 40 ml will be taken. In the event of unexplained abnormal laboratory test values, the tests will be repeated immediately and followed-up until they have returned to the normal range and/or an adequate explanation of the abnormality is found. If lab results haven’t returned to normal by the time of the next dose, vaccination will be suspended for that individual.

**Figure 1:** A schematic diagram of trial design and procedures

**Volunteers aged 18~45.**

**Written Informed Consent**

**Tests at screening**

**Testing:**

**Required test results**

**Physical examination**

**Inclusion/exclusion criteria**

**Eligible volunteers**

**Randomization within 5 groups, 4 verum, 1 comparator**

**Vaccination**

**Dose 1 of Grp 2- 50 ug/dose product**

**Dose 1 of Grp 3--100ug/dose product**

**Dose 1 of Grp 1-20ug/dose product**

| **Dose 1 of Grp 4--200ug/dose product;** |
| --- |

**AEs**

**Immunogenicity**

**Lymphocyte stimulation indices**

**Inhibition of parasite growth by immune sera**

Followed by dose 2 and 3 of the respective vaccine concentrations

**Data collection and analysis, written report**

## 5.3. Description of the measures taken to minimize/avoid bias:

The study will be conducted in a randomized fashion. The selection of volunteers will be based on inclusion and exclusion criteria prior to randomization. The volunteers will be randomly assigned to treatment groups according to the randomization list.

**5.4. Description of the investigational products**

The product information is seen in Background Information of the Investigation Brochure.

### 5.4.1 Supply and storage of the investigational vaccines

PfCP-2.9 vaccine formulation was produced in Shanghai Wanxing Bio-Pharmaceutical Company. The vaccine emulsion is packaged into autoclaved 2ml bottle with 1ml volume of emulsion, which contains various concentration of the vaccine antigen, i.e. 40, 100, 200 and 400 μg/ml. The vaccine emulsion is stored at 4℃ and has been shown to be stable for at least nine months. It is stored in a locked fridge with limited access only by a pharmacist who is in charge of the vaccine storage. The temperature is recorded on a daily basis.

### 5.4.2 Accountability procedures for the investigational product (including the Comparator).

Administration of the vaccine will be done under supervision of the investigators, by well- trained vaccinators from the local clinic. *Investigational Products Accountability Log* will be maintained in which an account of all products received, administered to volunteers, left over, lost, damaged or destroyed will be kept on a daily basis.

The principle investigator (PI) will account for all remaining investigational products at the end of the study (i.e. empty or partially used vials of stock solution *Product*, empty or partially used vials of reconstituted solution of *Product dilutions* or Comparator).

The PI must sign an acknowledgement for all supplies received. This acknowledgement will indicate the conditions of receipt of the supplies (physical intactness of package, reading of the cold chain indicator). Once the study report is prepared and with the sponsor’s authorization, the remaining products will be destroyed on site. A certificate of destruction will be issued and signed by the PI. These signed documents will be reconciled with delivered against used and destroyed stocks. The PI will have to be accountable for/explain any discrepancy. A pharmacist will be in charge of vaccine storage and accountability.

## 5.5 Description of the sequence and duration of the trial periods including follow-up and expected duration of volunteer participation

### 5.5.1 Duration of the study

(1). Recruitment: 6 months

(2). From the first vaccination to end of subjects’ follow-up: 8 months

(3). Final report writing: 2 months

TOTAL: 16 months

### 5.5.2 Expected duration of volunteer participation

The longest expected duration for volunteers is 10 months.

## 5.6 Identification numbers

There will be two numbers used in the study, i.e. *screening number* and *randomization number*. These 2 numbers will be recorded on the CRF.

The screening number will be given to the volunteers at the first screening visit. It will be composed of 3 digits starting with 001 and will be given to the subjects in a sequential fashion.

The randomization number will be given to the eligible volunteers whose conditions match all inclusion and none of the exclusion criteria, prior to vaccination on Day 0. The randomization number will be composed of 3 digits starting with 801 and will specify treatment code. The data management team will provide the list of randomization number and treatment code in advance. The initials, screening number and the randomization number will be written by the vaccine injector on the “Vaccine Information and Administration Form” accompanying each treatment vial.

## 5.7 Description of "discontinuation criteria" for individual volunteers and for the trial

### 5.7.1 For individual volunteers

An individual volunteer will be discontinued from any trial activities for any of the following reasons:

#### Withdrawal of consent

#### Presence of any abnormal medical condition, judged by the investigator medical team in consultation with the sponsor’s medical consultant

#### A severe or serious adverse event considered related to vaccination

All safety data prior to withdrawal of a volunteer will be collected and used in safety analysis if the volunteers are injected with the vaccine or Comparator. No withdrawn volunteers will be replaced in this study. After withdrawal or discontinuation, the investigator’s medical team should provide the best-proven treatment at no cost for any trial associated conditions that the volunteers have. Additionally, the team should carry out all safety and efficacy assessments that would have been carried out at the next scheduled visit (unless the volunteers are lost to follow up or withdraw their consent). The Study Conclusion Page in the CRF must be completed and the study investigational product records should be brought to up-to-date as far as possible. Choices of appropriate treatments will be under justification of the investigator’s medical team, with consideration of laboratory results. Similarly, no clinical evaluation per protocol will be further carried out in the withdrawn volunteers after the *Study Conclusion Page* (in CRF) has been completed.

**5.7.2 Study Amendment / Termination**

***Study amendment***

In the event that an induration or an ulcer measuring> 12 cm in diameter, lasting 48 hours, is observed in **any one** subject, the dose of the vaccine immediately lower will be used for the rest of the cohort, the DSMB will be consulted and the protocol amended accordingly.

**Go / No-Go criteria for Phase II**.

Further trials of the product will proceed only if at least 30% of participants have any antibody or cellular responses to the vaccine, with at least one dose- and adjuvant- regimen. The dose and adjuvant of choice for further trials will be selected on the basis of both safety profile and immunogenicity. The main immunological outcome will be the antibodies to PfCP-2.9. A regimen which leads to a response in a large proportion of the volunteers will be preferred to one with which the responses are of greater magnitude but present in fewer individuals.

## Study termination

The clinical monitor and the DSMB will review the safety profile of the vaccine formulation if one SAE attributable to the vaccine or >2 severe AEs occur. After discussion with the Investigators it will be decided whether to proceed with further injections, with higher, equivalent or lower doses, or to terminate the study.

DSMB reserves the right to discontinue the study at any time. The reasons must be discussed with the principal investigator and the Ethics Committee informed.

## 5.8 Minimum information to be recorded in the source documents

Source data should contain at least the following elements:

- Trial identification

- Volunteer initials

- Volunteer screening number

- Volunteer randomization number

- Items regarding inclusion/exclusion criteria

- Dates of visits and dates when volunteer stopped from the study

- AEs

- Concomitant medications/treatments/vaccinations

- Laboratory results (chemistry, hematology, immunology)

## 5.9 Identification of data to be recorded directly on the Case Report Form (CRFs)

All data will be recorded on the source data before being transcribed into the CRFs by the authorized study staff and kept updated at each visit.

## 6. Inclusion and exclusion criteria for vaccination

## Volunteer inclusion criteria for vaccination

### Healthy male and female 18 and 45 years of age

### Subjects who agree not to donate blood during the course of the trial.

### Signed written informed consent provided.

## 6.2. Volunteer exclusion criteria for vaccination

Prospective volunteer will be excluded from the study for any of the following reasons:

- History of allergic reactions or convulsions following vaccination.
- Involvement in drug or other vaccine trial within four weeks prior to the trial.
- Acute illness within four weeks prior to the trial.
- Presence of fever at the time of vaccination, i.e. body temperature (by mouth) > 37.5°C.
- Presence of any chronic illness/disease including diabetes mellitus, tuberculosis, leprosy, epilepsy and hypertension determined by medical history or examination.
- Persons on systemic corticosteroids, immunomodulators or anticoagulants within four weeks prior to vaccination.
- Persons with a history of allergic manifestations requiring treatment with injectable antihistamines adrenaline or steroids.
- Pregnancy. Women should not be pregnant and/or lactating and/or planning pregnancy throughout the study period. A urinary pregnancy test (immuno-chromatography) will be performed for all women of childbearing potential at entry and prior to each booster vaccination. Adequate contraception throughout the study should be used if applicable.
- Sexually active woman not using contraceptives.
- Smoker （≥20 cigarettes/day）.
- History of malaria: persons infected with malaria or with positive markers for antibodies to malaria parasite by IFA.
- History of ever traveling to or residing in a malaria endemic region or malaria exposure within last two years.
- Abnormal hematology and clinical chemistry (See Sections 8.1.2.1 and 8.1.2.2 for normal ranges of laboratory values).
- Persons with positive markers for HBV (HBsAg), HCV infection.
- ANF > 160.

## 6.3. Vaccination

The volunteers will be randomized to receive one of the following *5* regimens

### Group 1: 20 μg of the vaccine emulsion

### Group 2: 50μg of the vaccine emulsion

### Group 3: 100μg of the vaccine emulsion

### Group 4: 200μg of the vaccine emulsion

Group 5**:** Comparator

Vaccination will be administered *intramuscularly* in the left or right lateral deltoid*.*

## 6.4. Other medication(s)/treatment(s) authorized (including rescue medication) and not authorized before and/or during the trial.

### Medication(s)/treatment(s) NOT authorized during the trial

Steroids, immunomodulators, anticoagulants and other immunosuppressive drugs use are not authorized from one month prior to vaccination and throughout the period of the study.

Likewise, other vaccinations are not allowed from a period starting one month prior to vaccination and throughout the period of the study.

### Medication(s)/treatment(s) authorized during the trial

Volunteers are free to access any other necessary medications/treatments. However, they are required to inform the principal investigator in the event of receiving any form of medication or therapeutic intervention during the period of the trial.

# 7. Trial procedure

## 7.1 Recruitment and screening

### Recruitment

Prior to starting the study, workers in factories, students in universities and residents in Shanghai will be invited to attend an information meeting to describe the clinical trial and the role of volunteers. This will occur one month before the expected start of the study. This period will be needed to complete the whole process of recruitment.

### Screening (needing 6 months)

Within 6 month before the enrolment of the first group for vaccination, all individuals who agreed to participate will undergo the screening procedures. They will first sign informed consent form for the study. If the subject cannot read or write, a witness independent of the investigator team will sign the consent after oral consent has been obtained from the volunteer.

Inclusion and exclusion criteria will be checked.

A screening number will be given to the volunteers (3 digits in a sequential fashion).

The volunteers will be physically examined by one of the study investigators.

Screening procedures will be performed

About 70 individuals will be screened in order to allow recruitment for 52 volunteers. The screening will be performed as follows (for example):

A urinary pregnancy test will be performed for women of child bearing potential. Women with a positive pregnancy will be excluded from the study at this time. The volunteers will then be physically examined by one of the study investigators; blood pressure and body temperature (by mouth) will be recorded. A sample of 20 ml of venous blood will then be collected: 10ml for hematology and biochemistry analyses and 10 ml for immunological analyses. The biochemistry and hematology samples will be analyzed in clinical laboratory*.*

Blood samples will be labeled with the following information: trial code / volunteer screening number and initials / time point of the study / analyses to be performed.

The source document will be completed (including treatment and medical history at baseline).

## 7.2. Day 0 / Vaccination

Inclusion and exclusion criteria will be checked. All laboratory tests will be repeated again to ensure that the values are within the normal ranges. Females will be given a urine pregnancy test. Volunteers will be physically examined by one of the study investigators. Blood pressure and body temperature will be recorded

The volunteers whose conditions match all the inclusion and none of the exclusion criteria will be assigned a randomization number. For volunteers excluded for whatever reasons, results of analyses will be kept with the study documents. As shown in the safety Flow Chart (see below), on day 0, 3 verum and 1 placebo volunteers in group 1 and 5 will be injected intramuscularly in the left lateral deltoid with 0.5ml of adjuvant emulsion with 20μg and no antigen respectively. After two weeks, following a review of safety data associated with the first group, the remaining volunteers (7 verum and 2 placebo) from group 1 and 5 will be similarly injected intramuscularly. After 2 weeks of follow-up of this second cohort of group 1, the DSMB will review the cumulative safety (safety Assessment table sees appendix 4) for the entire group 1 and recommend whether it is safe to proceed to the next higher dose. PI must wait for a written reply from the DSMB before proceeding to the higher dose group. The volunteers in Group 2 will be immunized in a similar staged manner and will be injected with 50 μg of PfCP-2.9 in 0.5ml of adjuvant emulsion or with adjuvant emulsion alone in the left deltoid. Correspond to the process described above; three volunteers in group 2 and one in group 5 will be injected with the emulsion containing 50 ug and no antigen respectively. After two weeks, following a review of safety data, the remaining volunteers (7 verum and 2 placebo) from group 2 and 5 will be similarly injected. After two weeks of follow-up of this second cohort of group 2, the DSMB will review the cumulative safety data before proceeding to the next higher dose. The same process will be followed for group 3 (100 ug) and 4(200 ug). These injections will be repeated on Day 60 in the right lateral deltoid and Day180 in the left one in the same staged manner as first injection.

After the vaccination the volunteers will be led to the observation room for the monitoring of any possible acute AEs for 24 hour under the supervision of trained study staff who can identify and treat immediate adverse events (IAEs). Special attention will be paid to immediate hypersensitivity reactions.

**Figure 2: SAFETY FLOW CHART**

First Injection Group 1 Day 0

Verum (n=3) Placebo (n=1)

Group 1 group 5

im 20μg vaccine in 0.5ml

0.5ml placebo

2 weeks

Week 2 Evaluation of adverse events

Unacceptable Only acceptable adverse events

Adverse events

STOP Start the remaining volunteers in the Groups at Day 0

Verum (n=7) Placebo (n=2)

Group 1 group 5

0.5ml placebo

im 20μg vaccine in 0.5ml

2 weeks

Week 4 DSMB reviewing adverse events

Unacceptable only acceptable adverse events

Adverse events

STOP Start Group 2 at Day 0

Verum (n=3) Placebo (n=1)

Group 2 group 5

0.5ml placebo

im 50μg vaccine in 0.5ml

2 weeks

Week 6 Evaluation of adverse events

Unacceptable only acceptable adverse events

Adverse events

STOP Start the remaining volunteers at Day 0

Verum (n=7) Placebo (n=2)

Group 2 group 5

0.5ml placebo

im 50μg vaccine in 0.5ml

2 weeks

Week 8 DSMB reviewing adverse events

Unacceptable only acceptable adverse events

Adverse events

STOP Start Group 3 at Day 0

Verum (n=3) Placebo (n=1)

Group 3 group 5

im 100μg vaccine in 0.5ml

0.5ml placebo

2 weeks

Week 10 Evaluation of adverse events

Unacceptable only acceptable adverse events

Adverse events

STOP Start the remaining volunteers at Day 0

Verum (n=7) Placebo (n=2)

Group 3 group 5

0.5ml placebo

im 100μg vaccine in 0.5ml

2 weeks

Week 12 DSMB reviewing adverse events

Unacceptable only acceptable adverse events

Adverse events

STOP Start Group 4 at Day 0

Verum(n=3) Placebo(n=1)

Group 4 group 5

0.5ml placebo

im 200μg vaccine in 0.5ml

2 weeks

Week 14 Evaluation of adverse events

Unacceptable only acceptable adverse events

Adverse events

STOP Start the remaining volunteers at Day 0

Verum (n=7) Placebo (n=2)

Group 4 group 5

im 200μg vaccine in 0.5ml

0.5ml placebo

2 weeks

Week 16 DSMB reviewing adverse events

Unacceptable only acceptable adverse events

Adverse events

STOP

The source document and the Case Report Form (CRF) will be completed, including the screening section.

## 7.3. Follow-up visits (see Table 1)

After each vaccination, the volunteers will be discharged from the hospital. They will be given a Diary Card on which to record temperature and any local or systemic event occurring from time of vaccination and 14 days following vaccination, as well as any medication/treatment taken during this time and they will be asked to bring this Diary Card back at each visit. If a volunteer cannot read or write, another person from his/her household members will be asked to complete the diary.

The volunteers will be asked to come back to the trial site for follow-up on Days 1, 2, 7, 14, 30, 60, 61, 62, 74, 90, 180, 181, 182, 194 and 240. In case a volunteer does not appear for a follow up visit at the clinic, the study team will repeat telephone calls or eventually visit him/her at home. At each visit, AEs and SAEs will be checked and assessed. A physical examination will be performed including oral body temperature. Temperature will also be measured in case of suspected fever. Blood pressure will be measured at the visit. Similar to the assessment process on Day 0, the safety assessment will be emphasized on possible local and systemic AEs i.e. 1) for local AEs: pain, nodule, induration, erythrema, ulceration and itching, 2) for systemic AEs: fever, axillary and cervical lymphadenopathy, rash, nausea and vomiting. 3) Safety lab tests. In addition, any other symptoms volunteered by the vaccinees spontaneously will be recorded.

Volunteers with any clinical or laboratory abnormality will be followed up until they return to normal condition. Necessary treatments will be provided by the study medical team at no cost for any volunteers suffering from AEs. Any concomitant medication/treatments will be recorded and updated at each follow up visit.

The source document and the Case Report Form (CRF) will be completed at each visit.

**Table1. Activity schedule.**

| **Activities** | **Day** | | | | | | | | | | | | | | | | |
| --- | --- | --- | --- | --- | --- | --- | --- | --- | --- | --- | --- | --- | --- | --- | --- | --- | --- |
|  | **Screening** | **0** | **1** | **2** | **7**  **± 1** | **14**  **± 1** | **30**  **± 1** | **60**  **± 1** | **61**  **± 1** | **62**  **± 1** | **74**  **± 1** | **90**  **± 1** | **180**  **± 1** | **181**  **± 1** | **182**  **± 1** | **194**  **± 1** | **240**  **± 1** |
| **Preliminary information session, recruitment** | **X** |  |  |  |  |  |  |  |  |  |  |  |  |  |  |  |  |
| **I/E criteria veri-**  **fication** | **X** | **X** |  |  |  |  |  |  |  |  |  |  |  |  |  |  |  |
| **Urinary pregnancy test (1)** | **X** | **X** |  |  |  |  |  | **X** |  |  |  |  | **X** |  |  |  |  |
| **Interview / PE (2)** | **X** | **X** | **X** | **X** | **X** | **X** | **X** | **X** | **X** | **X** | **X** | **X** | **X** | **X** | **X** | **X** | **X** |
| **Informed Consent for vaccine study obtained** | **X** |  |  |  |  |  |  |  |  |  |  |  |  |  |  |  |  |
| **Blood sample for hematology/clinical chemistry** | **X** | **X** |  |  |  |  | **X** |  |  |  |  | **X** |  |  |  |  | **X** |
| **Blood sample for immunology** | **X** |  |  |  |  |  | **X** | **X** |  |  |  | **X** | **X** |  |  | **X*** | **X** |
| **Vaccination (3)** |  | **X** |  |  |  |  |  | **X** |  |  |  |  | **X** |  |  |  |  |
| **SAE reporting** | | | | | | | | | | | | | | | | | |
| **Concomitant treatment / vaccine recording** | | | | | | | | | | | | | | | | | |

**Notes:** I/E criteria =Inclusion/exclusion criteria, PE = Physical examination

(1) The pregnancy test will be performed prior to the blood sampling. In case of a positive pregnancy test, the volunteer will not undergo the remaining of the screening procedure or receiving further dose of vaccination.

(2) Physical examination includes adverse events = AE assessment (after vaccination) and temperature measurement. In addition, temperature should be measured every morning up to 10 days after vaccination and in case of suspected fever

(3) Volunteers will be asked to stay at the clinic for 24 hour following vaccination for monitoring of any possible acute adverse events

* 40 ml of blood will be collected. Parasie Inhibition test will be performed from serum collected at this time-point

## 7.4 Procedures for monitoring volunteer compliance.

The study officers will administer the vaccine injections. They will record the date of vaccination on the Case Report Form (CRF). At each visit, volunteers will be explained the study procedures for the next visit. The study team will make every effort to maintain the follow up record. Volunteers will be asked to refrain from taking drugs other than those permitted in the study. Each volunteer will be given an identification card to take with them at home. This card will inform other health care providers to consult the study medical team in case the volunteer presents to their health facilities with any ailment.

If a volunteer does not present to the clinic for a scheduled visit, the study team will contact him/her by telephone and visit him/her at residence on the next day. In case of loss to follow up, an active search will be made by the investigators to locate any volunteer lost to follow up and determine his/her health status. The investigator will document the search and its outcome.

**7.5 Contraception methods**

Women should avoid becoming pregnant for at least 4 months after vaccination. To avoid becoming pregnant, they will be advised to either abstain from sexual relations or practice a method of birth control. They will be made aware that no birth control method including the use of condoms, a diaphragm or cervical cap, birth control pills, IUD, or sperm killing products, is completely effective in preventing pregnancy. The Investigators will advise women about the most appropriate contraception method, if the volunteer is not already using a birth control method. Expenses related to contraception will be covered by the compensation given to all volunteers.

# 8. Assessment of Safety

## 8.1 Materials and Methods

### Clinical assessment

Clinical adverse events (AEs) including abnormal findings from the physical examination (such as blood pressure and body temperature abnormal values) during the trial will be recorded.

For each AE the following items will be collected and recorded in the CRFs:

- Symptoms
- Date of onset and date of end
- Intensity
- Relationship to trial vaccine (local adverse events at the vaccine injection site are considered related to the vaccine, if no injury has been experienced at the site caused by other causes)
- Severity
- Action taken

AEs assessment will be separated into local and systemic AEs and will be emphasized as follows. Between two planned visits subjects will be asked to complete a Diary Card to record any local or systemic adverse event(s) and any medication taken. At each planned visit, a physical examination will be performed under the responsibility of the PI.

***Local AEs***

**Pain**: The intensity of pain will be graded at 4 levels as follows:

Grade 0 = none

Grade 1 = mild: spontaneous pain and well tolerated

Grade 2 = moderate: pain-restricting movements

Grade 3 = severe: pain restricting normal daily activity

**Itching**: The intensity of itching will be graded at 4 levels as follows:

Grade 0 =none

Grade 1 = mild: itching at the site of injection and well tolerated

Grade 2 = moderate: itching at the site of injection, which is sufficiently discomforting to interfere with daily activities

Grade 3 = severe: itching at the site of injection that prevents normal daily activity

**Lymphadenopathy:** The intensity of lymphadenopathy will be graded at 4 levels as follows:

Grade 0 = normal

Grade 1 = Mild: lymph node ≤ 2 cm. Minimal tenderness (tolerated)

Grade 2 = Moderate: lymph node > 2 cm or moderate tenderness (interfering with daily activity)

Grade 3 = Severe: lymph node > 2 cm severe tenderness (preventing daily activity)

**Induration, Nodule, Ulceration***:* at the site of vaccination will be measured in cm with a graded ruler.

The maximum size will be recorded in the CRF.

***Systemic AEs***

**Fever:** The intensity of fever will be graded at 4 levels as follows:

Grade 0 = normal

Grade 1 = Mild: temperature (by mouth) ≥ 37.5°C but < 38°C

Grade 2 = Moderate: temperature (by mouth) ≥ 38.1°C but < 39.5°C.

Grade 3 = Severe: temperature (by mouth) ≥ 39.5°C

**Diarrhea:** The intensity of diarrhea will be graded at 4 levels as follows:

Grade 0 = normal

Grade 1 = mild: increase of 2-3 stools/day over pre-Rx

Grade 2 = moderate: increase of 4-9 stools/day, or nocturnal stools, or moderate cramping

Grade 3 = severe: increase of ≥10 stools/day, or grossly bloody diarrhea, or need for parenteral support

**Baldness:** The intensity baldness of will be graded at 4 levels as follows:

Grade 0 = normal

Grade 1 = mild: mild hair loss

Grade 2 = moderate: pronounced, or total hair loss and regenerate

Grade 3 = severe: total hair loss and no regeneration

**Anorexia:** The intensity anorexia of will be graded at 4 levels as follows:

Grade 0 = none

Grade 1 = mild: intake decreased less than 50%

Grade 2 = moderate: intake decreased more than 50% and need fluid infusion sometimes

Grade 3 = severe: no significant intake and fluid infusion is necessary

**Blood pressure** (lying position, after 5 minutes):

Hypertension:

Grade 0 = normal

Grade 1 = Mild: Transient increase > 10 mmHg diastolic pressures compared to screening value

Grade 2 = Moderate: Recurrent increase ≤ 20 mmHg and > 10 mmHg diastolic pressures compared to screening value

Grade 3 = Severe: Increase > 20mmHg diastolic pressure compared to screening value

Hypotension:

Grade 0 = normal

Grade 1 = Mild: Transient orthostatic hypotension with heart rate increased by > 20 beats/min OR systolic BP decreased by > 10 mm Hg, no Rx required

Grade 2 = Moderate: Symptoms OR BP decreased by > 20 mm Hg systolic, correctable with oral fluid Rx

Grade 3 = Severe: IV fluid required or hospitalization

**Other AEs (Rash, Vomiting, Nausea and etc)**

For other AEs, the grading system will be justified as shown below:

Grade 0 = normal or none

Grade 1 = mild: AE, which is easily tolerated

Grade 2 = moderate: AE, which is sufficiently discomforting to interfere with daily activities

Grade 3 = severe: AE, which prevents normal daily activity

**Other scalings**

*Action taken****:***0 = no, 1 = yes

*Seriousness*: 0 = non-serious, 1 = serious

*Relationship to vaccine:*

**A=Definite:** The experience:

- Follows a reasonable temporal sequence from the time of product administration;
- *And/or* follows a known response pattern to the trial product;
- *And* could not have been produced by other factors such as the patient’s clinical state, therapeutic intervention or concomitant therapy;
- *And* either occurs immediately following trial product administration, or there is positive reaction at the application site.

**B= probable:** The experience:

- Follows a reasonable temporal sequence from the time of product administration;
- *And/or* follows a known response pattern to the trial product;
- *And* could not have been produced by other factors such as the patient’s clinical state, therapeutic intervention or concomitant therapy.

**C=Possible:** The experience:

- Follows a reasonable temporal sequence from the time of product administration;
- *And/or* follows a known response pattern to the trial product;
- *But* could have been produced by other factors such as the patient’s clinical state, therapeutic intervention or concomitant therapy.

**D=Unlikely:** The experience was most probably produced by other factors such as the patient’s clinical state, therapeutic intervention or concomitant therapy, and does not follow a known response pattern to the trial product.

**E=Not related:** The experience is clearly related to other factors such as the patient’s clinical state, therapeutic intervention or concomitant therapy.

### Laboratory assessment

All the hematological and biochemical lab tests will be carried out in the Clinical laboratory of Changhai Hospital in Shanghai.

#### Clinical chemistry

Clinical chemistry will be evaluated at screening and on Day 0, 30, 90, 240 after vaccination including:

| **Test** | **Normal Range** |
| --- | --- |
| Blood creatinine level | 50 to 110 μmol/L |
| AST | < 40 u/L |
| ALT | < 50 u/L |
| Urine Sedimentation (12 hrs) | RBC< 500,000  WBC<1,000,000 |

The clinical chemistry laboratory analysis will be repeated during the follow up if any abnormality is found and will be repeated until values return to normal.

Clinical chemistry abnormality will be evaluated using the CTC grading (Appendix 1).

#### Hematology

Hematology will be evaluated at screening and on Days 0, 30, 90 and 240.

| **Test** | **Normal range** |
| --- | --- |
| Haemoglobin | Male: 12 to 16 g/dl  Female: 11 to 15 g/dl |
| Red blood cells | Male: 4.0 to 5.5 x 10 ^12^ / L  Female: 3.5 to 5.0x 10 ^12^ /L |
| Platelets | 100 to 300 x 10 ^9^ /L |
| White blood count | 4.0 to 10.0 x10^9^/L |
| Differential count:  Granulocytes (%)  Lymphocytes (%)  Monocytes (%)  Eosinophils (%)  Basophils (%) | 50 to 70  20 to 35  3 to 8  Up to 3  Up to 1 |

Hematology assessment will be repeated during the follow up if any abnormality is found and will be repeated until values return to normal.

Hematological abnormality will be evaluated using the CTC grading (Appendix 1).

# 8.2 Adverse events (AES) reporting

## 8.2.1 Definition

SAE is any untoward medical occurrence that at any dose:

- Results in death
- Is life-threatening
- Requires inpatient hospitalization or prolongation of existing hospitalization
- Results in persistent or significant disability/incapacity
- Or is a congenital anomaly/birth defect.

Medical and scientific judgement should be exercised in deciding whether expedited reporting is appropriate in other situations, such as important medical events that may jeopardize the patient or may require intervention to prevent one of the outcomes listed in the definition above. These should also usually be considered serious.

**8.2.2 Reporting and management of SAEs**

All serious adverse events (SAE) shall be reported within 24 hours by phone, fax or email to 1) the Sponsor 2) IRB/Ecs, 3) the Clinical Coordinator, 4) the Project Manager 5) the Clinical Monitor, and 6) DSMB, and 7) SDA. This will be followed, within 5 days, by a written report that gives additional information (use SAE report form) including a description of the SAE, onset, date and type, duration, intensity, cause-effect relationship with the vaccine, outcome, measures taken (symptomatic treatment) and all other relevant clinical and laboratory data. The SAE, whether vaccine-related or not, will be followed until resolution.

Trial may be put by the DSMB on hold until permission or otherwise is given by the DSMB, the Local Ethics Committee and SDA.

All AEs (including laboratory abnormalities) will be recorded in the CRFs. After the trial has been completed or terminated, all recorded AEs will be listed, evaluated and discussed in the final report.

## 8.3 Criteria for safety

Safety will be evaluated by the incidence, intensity, seriousness and relationship of new adverse event(s), and clinically relevant changes as described in Section 8. Safety profile of the vaccine will be evaluated using the same criteria. The vaccine will be considered ***safe*** if:

(i) There is no serious adverse event attributable to the study vaccine

(ii) Incidence of any adverse event attributable to the study vaccine with grade 3 is not greater than 6%, i.e. ≤3 out of 52 subjects.

# 9. Assessment of immunogenicity

## 9.1 Materials and Method for the assessment of the immunogenicity endpoints

All immunological analyses will be performed in agreement with SOPs available on site.

IFAT to test for antibodies to blood stages using cultured parasite.

ELISA for the determination of IgG to PfCP-2.9

Cellular Immunology: Lymphocyte stimulation tests to PfCP-2.9

Ability of total Ig G from immune sera to inhibit growth of *P. falciparum* in vitro.

IFAT, ELISA and Parasite inhibition assay will be performed in the Molecular Vaccine Development Laboratory in Secondary Military Medical University, Shanghai but the Lymphocyte Stimulation test will be done by the hematological laboratory of Changhai Hospital.

Upon completion of each cohort, all specimens for one vaccine will be treated on the same day. On completion of the follow up, all samples will be repeated at the same time for ELISA determination of IgG to PfCP-2.9 and the results obtained will be used for immunogenicity analysis.

9.2 Criteria for immunogenicity

*Antibody titres* as determined by ELISA to PfCP-2.9

*T cell stimulation indices* determined using PfCP-2.9 to stimulate PBMCs

*The proportion of responders to PfCP-2.9*, responders being defined as

(a) Antibody responder: participant whose post immunization serum has a titre of greater than 1:500

(b) High antibody responder: participant whose post immunization serum has a titre of greater than 1:10,000.

(c) Lymphocyte responder: participant whose lymphocyte stimulation indices are greater than 3.

An individual may be both an antibody and lymphocyte responder.

*Sera* *inhibit rates* as determined by *in vitro* inhibition assay using *Plasmodium falciparum isolate* FCC1/HN and 3D7.

# 10. Statistical analysis

Safety data: Vital signs collected at baseline and on Days 1, 2, 7, 14, 30, 60, 61, 62, 74, 90, 180, 181, 182, 194 and 240 will be listed for the study group.

Adverse Events: Descriptive statistics will be used to analyse adverse events (AEs), including intercurrent illnesses, for the study group. The number of AEs and their severity will be assessed. Safety data will be reported using frequency tables of numbers of AEs. With frequently occurring (10 or more) event types, effects of adjuvant and vaccine dose will be tested using logistic regression models, with both the type of adjuvant and vaccine dose as factors in the model. Statistical significance will be assessed by likelihood ratio tests.

Immunogenicity Data: Immunological data for each time point will be analysed separately.

Descriptive statistics of antibody levels determined by the specific anti-PfCP-2.9 antibody titers at baseline and on days 0, 30, 60, 90, 180, 194 and 240.

Descriptive statistics of CD4^+^ T-cell proliferative response, expressed as stimulation index (SI) as determined by lymphocyte proliferation assay described in Appendix 2.

Assessment of new responders for humoral and cell-mediated immunity after each immunisation and overall: A “responder” is a subject that has a positive response, in the immunological tests:

For antibodies, the *baseline* is the value obtained in naïve subjects (before injections) at a dilution of 1/200. A positive antibody response is equal to the baseline + 3 standard deviations of the baseline measurements.

For cell-mediated immune responses (CMI), positive responses are as follows:

- - T-cell proliferation: SI > 3 (stimulation index)

Comparisons of the response rate for each immune response will be tested separately for each time point using logistic regression models. Both the type of adjuvant and vaccine dose will appear as factors in the model. Statistical significance will be assessed by likelihood ratio tests.

Comparison of logarithmically transformed values of each immune response variable will be carried out using two-way analysis of variance; again, both the type of adjuvant and vaccine dose will appear as factors in the model.

All statistical analyses tests will be performed at an individual significance level of 5% and will be two-sided and will be regarded as exploratory. Therefore no alpha-adjustment for multiplicity is needed.

In addition all data will be reported in patient listings.

## 10.1 Sample size calculation

## Usually Phase I trial enrolls 30-50 cases, divided into 3-5 groups, for safety assessment. The data from Phase I trial is usually analyzed in a descriptive statistic mode. The sample size for this study is determined by the requirement to determine safety of each dosing regimen. The study design cannot ensure that differences in immunogenicity between regimens are statistically significant. Ten subjects at each vaccine dose constitute a reasonable sample size to estimate the frequency of AEs with an acceptable accuracy.

## 10.2 Safety analysis

### 10.2.1 Methods for analyzing safety

The incidence of systemic AEs will be tabulated by intensity and relationship to trial vaccine. The number of volunteers with maximum intensity grade will be presented by treatment group in a table.

Incidence of local AEs will be tabulated by type and intensity.

SAEs will be described.

Abnormal laboratory values will be described.

### 10.2.2 Data sets to be analyzed for safety

Safety measurement for the vaccine will be summarized for the intention to treat volunteers, i.e., all randomized volunteers who were injected at least one dose of the test vaccine.

## 10.3 Immunogenicity analysis

### 10.3.1 Method for analyzing immunogenicity

### Immunogenicity analysis will be performed for blood samples taken at Screening (before vaccination), Day 30, 60, 90, 180, 194 and 240. Elisa, IFA and Lymphocyte stimulation assay will be used to assess immunogenicity. Parasite inhibition test will be done on Day 194 blood sample only.

### 10.3.2 Data sets to be analyzed for immunogenicity

Immunogenicity will be analyzed in evaluable volunteers. The volunteers are considered to be evaluable if they have received the vaccination and have completed the follow up visits up to Day *(D240)*, and have no protocol violation that could interfere with the interpretation of the results (such as intake of unauthorized drugs for instance).

A volunteer who has received vaccination and has withdrawn from the trial will be included in the analysis of safety.

## 10.4 Procedure for accounting for missing, unused, and spurious data.

Missing data will be coded with an appropriate code on the written and electronic records. Unused or inconsequential data will remain on the original source documents. Spurious data will be investigated and if corrections are made any modifications to the source data or CRF will be signed and dated by an authorized study staff member.

## 10.5 Procedures for reporting any deviation(s) from the original statistical plan

Any deviation from the approved statistical plan will be described and justified in the final report. In addition, it will be presented to the DSMB and the sponsor prior to the statistical analysis.

# 11. Direct access to source data/documents

The investigators will provide written agreement (see SOP CTO5) that the investigator(s) /institution(s) will permit trial-related monitoring, audits, IRB/IEC review, regulatory inspection(s) and providing direct access to source data/documents.

# 12. Quality control and quality assurance

The sponsor implements and maintains quality assurance and quality control systems with written SOPs to assure that trials are conducted and data are generated, recorded, and reported in compliance with the protocol, GCP, and the applicable regulatory requirement(s). Prior to the enrollment of any volunteer at a site, the clinical monitor and investigator will review the protocol and all trial related procedures. This includes information on the study medication, procedures for obtaining informed consent, procedures for reporting AEs and procedures for completing the CRFs. Site monitoring visits will be scheduled by the clinical monitor on a regular basis. During these visits, information recorded in the CRFs will be verified against source documents for accuracy and completion. The clinical monitor will review the informed consent procedure, product accountability and storage, trial documents and trial progress. The clinical monitor will verify that the investigator follows the approved protocol or amendments (if any), he/she will observe trial procedures and will discuss any problems with the investigator. Monitoring visits will be recorded in the *Monitoring Log* at the investigator’s site, and at the end of the trial a copy of the completed log will be returned to the sponsor.

Personal volunteer data will be kept confidential. The file cabinets with the trial data and the volunteer information will be locked and accessed by only the sponsor and Regulatory Authorities. CRFs or other documents submitted to the sponsor will identify a volunteer by the first initial of the first four names and by the randomization number only. The investigator will keep in the investigator’s files a *Subject Identification List* + *Screening/Enrolment Log* (including complete name, age and address). To allow compliance with GCP principles, each volunteer will be asked for consent regarding direct access to the source documents for monitoring, audit, and inspections. The agreement covering the use of the data or analysis has to be documented in writing, together with the written informed consent for trial participation.

## 12.1 Essential documents for trial initiation

The investigator has to provide to the sponsor prior to the initiation of the study the following “essential documents”:

1. Written ethics committee (IEC/IRB) note on the review of the clinical trial protocol and informed consent form (and of other trial related documents required locally)
2. Agreement on Study Conduct according to the protocol signed by the PI
3. Technical Services Agreement (TSA) signed by WHO/TDR/PRD and investigator/institution, if applicable.
4. Curriculum vitae of principal investigator and co-investigators.
5. Regulatory authority (ies) authorization/approval/notification of the trial
6. Current laboratory normal ranges and documentation of laboratory certification (if applicable).

The sponsor will maintain *Trial Master Files* (TMF) for the ‘essential documents’ as specified by ICH-GCP. The sponsor will provide the principal investigator with the *Investigator’s File*, containing several relevant documents. The investigator’s file will be kept and updated by the investigator, and checked by the clinical monitor. TMF will be updated by the sponsor.

## 12.2 Archiving

The investigator must arrange for the retention of the volunteer’s identification list for at least 15 years after completion or discontinuation of the trial. The investigator is required to retain all volunteer files and source documents for 10 years. The sponsor will inform the investigator(s) as to when these documents no longer need to be retained.

No document pertinent to the trial should be destroyed without prior written agreement between the sponsor and the investigator. Should the investigator wish to assign these records to another party, or move them to another location, written agreement must be obtained from the sponsor. The originals of protocol, CRFs, and *Product Accountability List* will be archived by the sponsor.

# 13. Data handling and management

All trial data will be recorded on the CRFs supplied by the sponsor. Only the investigator and authorized co-workers, according to the list of *Authorized Signatory Form (ASF)*, are authorized to make entries on the CRF. The CRF should be completed in English.

Entries into the CRF should be made with a black or blue ink ballpoint pen to ensure legibility. Corrections should be made by drawing a single line through the original entry, entering the new value and placing initials and date next to the new entry (*follow the procedure in SOP CT 05*). Completed CRFs will be dated and signed by the investigator or authorized study personnel.

Source documents including medical records and original laboratory results will be kept in a separate file at the investigator’s office. CRFs will be kept in a room with limited access.

After completion of the CRFs by the investigator, the clinical monitor will review all CRFs for completeness and accuracy before sending them to the clinical data manager. The data will be entered into a database where computer checks are used to identify selected protocol violations and data errors. Alternately, this can be done manually. Requests for clarifications or correction will be sent to the investigational team, if necessary. Data analysis will be carried out after all enquiries have been done and the database has been locked.

# 14. Ethical Aspects

## 14.1 Approval by the local IRB/IEC and SCRIHS

The protocol of this study will need approval from the local IRB/IEC and the Secretariat Committee for Research Involving Human Subjects (SCRIHS) of WHO before it is initiated. The Principal Investigators will submit the study protocol with all necessary attachments and documentation to these authorities. A copy of the written approval from the local ethics committees will be sent to the sponsor. The Project Manager will then submit the proposal to WHO/SCRIHS for final approval.

## 14.2 Volunteer Information and Informed Consent

a. Each volunteer will receive written information about the study, which will also be explained in the presence of a witness . Sufficient time will be given to the volunteer to decide whether or not to participate in the study.

b. Volunteers will be given the opportunity to enquire about the details of the study and any question regarding the study will be answered.

c. The PI will ensure that the consent forms have been signed and dated by the volunteer, physician, and independent witness.

e. The PI will ensure that the written information and the consent form are revised and signed when an amendment to the clinical trial protocol is made that will impact the volunteer’s decision to continue in the study and approved.

f. A copy of the volunteer information and consent form as used will be given to the clinical monitor.

Before entering the study, each volunteer will give written informed consent. Consent will be verbal for any volunteer who is illiterate. In this situation, an independent witness will be required who will then sign the informed consent form.

By signing this protocol, the PI assures the sponsor that informed consents will be obtained and that the consent form and consent information to be used will be submitted to the Ethics Committee responsible for approving the conduct of the study.

# 15. Financing and insurance

Financing and insurance will be addressed in a separate document.

# 16. Publication policy

It will be the responsibility of the PI to produce a report to the PRD/Product Development Team (PDT) for review and comment. The PI and the Project Managers will agree on the contents of any publication that will need to be cleared by the sponsor. The PIs agree not to publish or communicate the data in public without written authorization by the Project Managers.

Draft versions of abstracts or manuscripts must be made available to the co-authors and to the sponsor before presentation of results or submission for publication. At least 30 days should be allowed for review and comment of an abstract and full paper.

# 17. Protocol amendment

After the protocol has been signed, any amendment of the protocol has to be agreed between the investigator and the sponsor in the form of a written amendment. Any change will be signed and dated by the PI and the Project Manager/Clinical Coordinator and attached to the original protocol (following the procedure in SOP CT 05).

All amendments will be notified to the local IRB/EC. The IRB actually should have rules or SOPs that guide them as to which amendments require approval by the IRB prior to implementation of the amendment----some amendments can be considered ‘administrative’, i.e. does not affect the conduct of the trial or the volunteer. The amendment should also indicate whether there is the need for any modification to the informed consent form, CRFs etc. The investigator will acknowledge in writing receipt of the amendment.

# 18. Consent Form

A *Phase 1 randomized single center study*, comparing doses of PfCP-2.9 recombinant vaccine adjuvanted with Montanide ISA 720 *for* safety and immunogenicity

Study *S001* – version 04

# 19. Abbreviations

| AE | Adverse event |
| --- | --- |
| CRF | Case Report Form |
| EC | Ethics Committee |
| IFN-γ | Interferon gamma |
| IRB | Institutional Review Board |
| PBMCs | Peripheral blood mononuclear cells |
| PI | Principal Investigator |
| PRD | Product Research and Development |
| SAE | Serious Adverse Event |
| TDR | Special Programme for Research and Training in Tropical Diseases |
| WHO | World Health Organization |
| PfCP-2.9 | *Plasmodium falciparum Chimeric* Protein 2.9 |
| ELISA | Enzyme-linked immunoabsorbent assay |
| IFA | Indirect Immunofluorescence Assay |
| GIA | Growth inhibition assay |
|  |  |
|  |  |
|  |  |

# 20. References

1. Patarroyo ME et al, Induction of protective immunity against experimental infection with malaria using synthetic peptides, Nature, 328:629, 1987
2. Alonso PL et al, Randomized trial of efficacy of SPf66 vaccine against *Plasmodium falciparum* malaria in children in southern Tanzania, Lancet, 344:1175, 1994
3. D’Alessandro U, et al, Efficacy trial of malaria vaccine SPf66 in Gambian infants, the Lancet, 346:462,1995
4. Gordon DM et al, Safety, immunogenicity, and efficacy of a recombinantly produced *Plasmodium falciparum* circumsporozoite protein-hepatitis B surface antigen subunit vaccine, J Infect Dis, 171:1576, 1995
5. Kester KE et al, Efficacy of recombinant circumsporozoite protein vaccine regimens against experimental *Plasmodium falciparum* malaria, J Infect Dis, 183:640, 2001
6. Holder, A. A. 1988. The precursor to major merozoite surface antigens: structure and role in immunity. *Prog. Allergy* 41:72-97.
7. Holder,A.A.and Freeman,R.R.Immunization against blood-stage rodent ,malaria using purified parasite antigens.*Nature*,294,361-364,1981.

8. Daly TM et al,Comparison of the carboxy-terminal,cysteine-rich domain of the merozoite surface protein-1 from several strains of *plasmodium* *yoelii*.Mol.Bio.Parasit.52:279-282,1992

9. Blackmanm M J,et al,Proteolytic processing of the *Plasmodium falciparum* merozoite surface pro tein-1 produces a membrane-bound fragment containing two epidermal grow factor-like domains. Mol Biochem Parasitol, 49:29~33, 1991

10.TanabeK et al,Allelic dimorphism in a surface antigan gene of the malaria parasite *Plasmodium falciparum* .J.Mol.Bio.,195,273-287,1987

11. Miller,L.H.,Roberts,T.,Shahabuddin,M.and McCutchan,T.F.Anaalysis of sequence diversity in the *Plasmodium falciparum* surface protein-1 (MSP1).Mol.Biochem.Parasitol.,59,1-14,1993.

12. Kaneko,O.,Kimura,M.,Kawamoto,F.,Ferreira,M.U and Tanabe,K. *Plasmodium falciparum* allelic variation in the merozoite surface protein 1 gene in wid isolates from southern Vietnam. *Exp.Parasitol*.,86,45-57 ,1997.

13. QariSH et al,Predicted and observed alleles *Plasmodium falciparum* of merozoite surface proteins(MSP1),a potential malaria vaccine antigen.Mol.Bio.Parasit.92:241-152,1998

14. GarraaudO et al,Secretin of parasite-specific immunoglobulin G by purified blood B lymphocytes from immune individuals after in vitro stimulation with recombinant *Plasmodium falciparum* merozoite surface protein1-19 antigen.Immunology.96(2):204-10,1999

15.Holder,A.A.and Freeman,R.R.Immunization against blood-stage rodent ,malaria using purified parasite antigens.*Nature*,294,361-364,1981.

16. Siddiqui, W. A., L. Q. Tam, K. J. Kramer, G. S. N. Hui, S. E. Cade, K. M. Yamaga, S. P. Chang, E. B. T. Chan, and S.-C. Kan. 1987. Merozoite surface coat precursor protein completely protects *Aotus* monkeys against *Plasmodium falciparum* malaria. *Proc. Natl. Acad. Sci USA* 84: 3014-3018

17. Chang SP et al, A recombinant baculovirus 42-kilodalton C-terminal fragment of *Plasmodium falciparum* merozoite surface protein-1protects Aotus monkeys against malaria. Infect.and Immun.64(1):253-261,1996

18. Deans JA, et al, Vaccination of rhesus monkeys with a minor, invariant, Plasmodium knowlesi 66 kDa merozoite antigen. Parasite immune. 10:535-552,1988

19. Howell SA et al, Proteolytic processing and primary structure of *Plasmodium falciparum* apical membrane antigen-1. J Biol. Chemi. 276:31311, 2001

20. Lawrence G, Saul A, Giddy AJ, Kemp R, Pye D—“Phase I trial in humans of an oil-based adjuvant SEPPIC MONTANIDE ISA 720” Vaccine, Vol15, No 2, 176-178, 1997

21. Saul A, Lawrence G, Simllie A, Rzepezyk C, Reed C, Taylor D, Anderson K, Stowers A, Kemp R, Allworth A, Anders RF, Brown GV, Pye D, Schoofs P, Irving DO, Dyer SL, Woodrow GC, Briggs RS, Reber RO, Sturchler D—“Human Phase 1 vaccine trial of 3 recombinant asexual stage malaria antigens with Montanide ISA 720 adjuvant”. Vaccine 17, 3145-3159, 1999

22. Lawrence G, Cheng Q, Reed C, Taylor D, Stowers A, Cloonan N, Rzepczyk C, Smillie A, Anderson K, Pmbo D, Allworth A, Eisen D, Anders F, Saul A—“Effect of vaccination with 3 recombinant asexual-stage malaria antigens on initial growth rates of Plasmodium falciparum in non-immune volunteers.” Vaccine 2000 Mar 17; 18:1925-31.

23. Genton B, Al-Yaman F, Anders R, Saul A., Brown G, Pye D, Irving DO, Briggs WR, Mai A, Ginny M, Adiguma T, Rare L, Giddy A, Reber-Liske R, Stuerchler D, Alpers MP-“Safety and immunogenicity of a three-component blood-stage malaria vaccine in adults living in an endemic area of Papua New Guinea.” Vaccine 2000 May 22; 18 (23): 2504-2511

24. Genton B, Inoni Betuela, Ingrid Felger, Fadwa Al-Yaman, Robin Anders, Allan Saul, Lawrence Rare, Moses Baisor, Kerry Lorry, Graham Brown, David Pye, David Irving, Thomas Smith, Hans-Peter Beck, Michael Alpers “A recombinant blood-stage malaria vaccine reduces Plasmodium falciparum density and exerts selective pressure on parasite populations in a PhaseI/IIb trial in Papua New Guinea” (in press JID)

25. Lawrence, G (informant) et al. Phase 1 study with AMA-1 malaria antigen in Australia, (unpublished data, personal communication)

26. Duarte Cano CA---“The multi-epitope polypeptide approach in HIV-1 vaccine development.” Genet Anal 1999 Nov;15 (3-5):149-153.

27. Duarte Cano CA---“A Phase I clinical trial of a multi-epitope polypeptide TAB9 combined with Montanide ISA 720 adjuvant in non-HIV-1 infected human volunteers” Vaccine 19(2001) 4328-4336)

28. Lopez JA, Weilerman C, Audran R, Roggero MA, Bonelo A, Tiercy JM, Spertini F, Corradin G. “ A synthetic malaria vaccine elicits a potent CD8+ and CD4+ T lymphpcyte immune response in humans. Implications for vaccination strategies.” Eur. J. Immunol. 2001. 31: 1989-1998

# APPENDIX 1:

# Common Toxicity Criteria

| **TOXICITY** | **Grade** | | | | |
| --- | --- | --- | --- | --- | --- |
|  | **0** | **1** | **2** | **3** | **4** |
| HEMATOLOGY | | | | | |
| WBC  PLT  Hgo g/100ml  g/l  mmcl/l  Granulocytes/  Bands  Lymphocytes | >4.0  WNL  WNL  WNL  WNL  >2.0  >2.0 | 3.0 – 3.9  75.0 – normal  10.0 – normal  100 – normal  6.2 – normal  1.5 – 1.9  1.5 – 1.9 | 2.0 – 2.9  50.0 – 74.9  8.0 – 10.0  80 – 100  4.95 – 6.2  1.0 – 1.4  1.0 – 1.4 | 1.0 – 1.9  25.0 – 49-9  6.5 – 7.9  65 – 79  4.0 – 4.9  0.5 – 0.9  0.5 – 0.9 | <1  <25.0  <6.5  <6.5  <4.0  <0.5  <0.5 |
| CLINICAL CHEMISTRY | | | | | |
| Hypoglycemia | >64mg/dl  >3.6mmol/l | 55 – 64  3.1 – 3.6 | 40 – 54  2.2 – 3.0 | 30 – 39  1.7 – 2.1 | <30  <1.7 |
| Hyperglycemia | <116mg/dl  <6.2mmol/l | 116 - 160  6.2 - 8.9 | 161 - 250  9.0 - 13.9 | 251 –5 00  14.0 - 27.8 | >500 or ketoacidosis  >27.8 or ketoacidosis |
| Creatinine | WNL | <1.5 x H | 1.5 – 3.0 x H | 3.1 – 6.0 x H | >6.0 x H |

# APPENDIX 2:

# Procedures for the measurement of immunological markers

1. **This SOP describes the procedures for ELISA**

**A 、Principle:**

The key to the ELISA system is the specific reaction between antigens and antibodies.

Specific antibodies can be measured by the use of defined antigens. ELISAs make use of an enzyme attached to one of the reagents utilized in the test. Subsequent addition of suitable enzyme substrates causes a color change; the results can be read by specially designed spectrophotometers. Indirect ELISA is the most useful in the ELISAs to titrate antibodies against specific antigens. After antigens are passively attached to plastics (solid phase), specific antibodies can bind to the antigens and unreacted material can be simply washed away. The specific antiserum against the antigen is not labeled with an enzyme, but a second antibody specific for the particular species in which the first antibody was produced is labeled. It offers the advantage that any number of antisera can be examined for binding to a given antigen using a single antispecies conjugate. In this case, an optimal amount of PfCP-2.9(0.1 ug/well) is absorbed to wells, and anti-sera from immunized volunteers are diluted and added to each well. Any antigen-antibody reaction is then detected by addition of an optimal amount of antispecies conjugate. The antibodies titration of the specific antiserum will be taken as the highest serum dilution which gave an optical density (OD value) greater than twice that of pre-immune sera.

**B、 Materials required:**

Antigen: purified protein(1ug/ml);

Antibody: immunized volunteer’s sera;

Second antibody: goat anti-human IgG linked to horseradish peroxidase;

96-well microplates;

Multichannel pipettes;

10ul,100ul,1000ul pipettes;

Coating buffer: carbonate/bicarbonate buffer PH9.6, 0.05M;

Blocking buffer: PBS containing 3%nonfat-dried milk;

Washing buffer: PBS containing 0.05%Tween20;

PBS containing 3% nonfat-dried milk and 0.05%Tween20;

TMB solution;

2M sulfuric acid in water;

Microplate reader;

Wellwash machine;

Incubator,37℃;

Centrifuge tubes(1.5ml);

Paper towels

**C、Procedure:**

1. Add 100ul of a 1 ug/ml antigen solution diluted in coating buffer (carbornate/bicarbonete buffer ) to each well using a multichannel pipet.
2. Incubate at 37℃ for 1hr
3. Remove antigen solution by flicking , wash the wells in washing buffer and empty wells for three times.
4. Add 100 ul of blocking buffer to each well and incubate for 1hr a 37℃.
5. Make a five-fold range dilution of the test sera in 1.5ml tubes from tube 1 to tube 6. Add 1000ul of PBS containing 3% nonfat-dried milk and 0.05%Tween20 to tube 1 and 400ul to the rest, then add 2ul of test sera to tube 1 ,after mixing , transfer 100ul to tube 2,after mixing , transfer 100 ul to tube 3. In the same way for the rest tubes. Thus we have obtained a five-fold dilution range of the test sera.
6. Add 100ul of the diluted sera to 96-well plates, triplicates for each dilution.
7. Incubate the plate at 37℃ for 1 hr.
8. Remove the dilution, and wash the plate for four times.
9. Add 100ul of a 1:1000 dilution of peroxidase-conjugated horse anti-human IgG to each well .

11、Incubate at 37℃ for 1hr.

12、Remove the dilution, and wash the plate as above.

13、Add 100ul of TMB solution.

14、Incubate for 6~10 min at room temperature (note color changes).

15、Stop color development by adding 50ul of 2M sulfuric acid to each well.

16、Read the plate at 450/630nm by a Bio-Tek microplate Reader model ELX800.

17、Calculating the serum titration through comparing to the OD value of the control serum.

2. This SOP describes the procedures for Indirect fluorescence assay (IFA)

**A. Principle:**

As ELISA, IFA is used to detect the antibodies level in immunosera. The major difference between IFA and ELISA is that the IFA can quantify the antibodies which recoganize the natural antigens. Just as the capture antigen in ELISA ,the cultured malaria parasites, for example Plasmodium falciparum, are fixed on glass slides and used to detect corresponding antibodies through reacting with its surface antigens. When diluted sera are incubated with the slide carrying parasites, the specific antibodies recoganize nature antigens and bind to them. After washed, those nonspecific antibodies are washed away from the slide. We can detect the binding antibodies by the second antibodies as goat IgG against human IgG labeled with fluorescein isothiocyanate (FITC). After washed, the binding antibodies can be observed with fluorescent light microscopy. By this indirect way, we can gain the antibody level in the test serra via comparing with pre-immune sera.

**B. Material requested:**

Glass slide

1.5ml conical centrifuge tubes

Methanol

10mM pH7.4 PBS

Nonfat powdered milk

Fluorescein isothiocyanate(FITC)-conjugated goat IgG against human IgG

Antiserum

Plasmodium falciparum

Fluorescent light microscopy.

**C. Procedure**

1. Preparing thin blood smears using cultured Plasmodium falciparum.
2. Air-dried slide are fixed with ice-cold methanol for 15 minutes.
3. Washed with 10mM pH7.4 PBS.
4. Blocked with 3% nonfat powdered milk in PBS(blocking buffer).
5. The test sera was diluted with blocking buffer, and add them onto the slide.
6. Incubate the slide for 1 hour at 37℃.
7. Washed with PBS for three times, 5 minutes each time.
8. Dilute the FITC-conjugated anti-human IgG at 1:100 with blocking buffer, and add it onto the slide.
9. Incubate the slide for 1 hour at 37℃.
10. Washed with PBS as step 7.
11. Read the slide with fluorescent light microscopy. The End Point IFA titer is defined as the final serum dilution producing parasite immunofluorescence above background level.

**3. This SOP describes the procedures for *In vitro* inhibition assay**

**A. Principle:**

Invasion of merozoite into erythrocyte may be mediated by receptor(s) on the host cell and ligands on the parasite. Antibodies to the ligands may inhibit the merozoite invasion and the parasite growth. Continuous cultivation of several strains of *Plasmodium falciparum* has been established in our laboratory. Moreover we have set up an inhibition assay to evaluate abilities of either immune sera or specific antibody to inhibit parasite growth in vitro.

**B. Material requested:**

Strain: *Plasmodium falciparum* FCC1/HN (Hainan, China);

Plasmodium falciparum 3D7

Blood: A^+^ blood from standard blood bank in CPD-A1

Medium: RPMI1640 containing 15% human or rabbit sera

Pre-immune and immune rabbit sera

Giemsa solution

GNS solution:

CO_2_ incubator: adjust to 37℃, 5% O_2_ ,5% CO_2_

15ml conical centrifuge tubes

96-well microculture plate

**C. Procedure:**

1．Parasite cell preparation: the parasite is continuously maintained in Complete malaria culture medium using standard method.

2．Erythrocytes preparation: wash the blood with 5% GNS twice, draw off supernatant and add complete malaria culture medium to give 50% hematocrit ,stored at 4℃ for 1 week.

3．Synchronization of parasites: the parasite culture with a majority of rings are Synchronized by treatment with 5% sorbital .

1. Concentration of schizonts. late-trophozoite and schizont-stage Parasites were purified from synchronous cultures by centrifugation on 80-60-50-40% layered Percoll

5．Preparation of initial culture. The parasites are diluted with uninfected erythrocytes to give a parasiteamia of 0.3% and a hematocrit of 2% in ICM.

6．Add 200 ul of culture containing 30ul of test sera and 170ul initial culture in triplicate wells into 96-well plates

7．Incubated at 37℃ in the CO_2_ incubator for 72 h and the medium containing test sera is refreshed at 24h interval.

8.Thin smears were prepared from each well, fixed in methanol and stained with Giemsa’s solution for 30 min.

9．Parasitemia is determined by oil immersion light microscopy, The inhibition rate was determined according to the following: % inhibition = (Pc-Ps)-(Pt-Ps)/(Pc-Ps) x100%. Pc: parasitemia of preimmune sera; Pt: parasitemia of immune sera; Ps: parasitemia of starting culture.

**4). This SOP describes the procedures for lymphocyte proliferation assay**

**A. Principle:**

Peripheral blood is the primary source of lymphoid cells for investigation of the human immune system. Its use is facilitated by Ficoll-Hypaque density gradient centrifugation – a simple and rapid method of purifying peripheral blood mononuclear cells (PBMC) that takes advantage of the density differences between mononuclear cells and other elements of the blood sample. Mononuclear cells and platelets collect on top of the Ficoll-Hypaque layer because they have a lower density; in contrast, red blood cells (RBC) and granulocytes have a higher density than Ficoll-Hypaque and collect at the bottom of the Ficoll-Hypaque layer. Platelets are separated from the mononuclear cells by subsequent washing or by centrifugation through a fetal-calf-serum cushion gradient, which allows penetration of mononuclear cells but not platelets. The mononuclear cell sample can be purified in the following protocols.

Ficoll-Hypaque gradient centrifugation allows rapid and efficient isolation of mononuclear cells from human peripheral blood (ref). As such, this technique is the starting point of most studies of human lymphoid cells. In general, Ficoll-Hypaque centrifugation does not change either the phenotype or the function of the isolated mononuclear cell population.

Lymphocyte proliferation and secretion is an important stage in immune reaction, so proliferation assays are widely used to assess the cell immunocompetence. T cell receptor(TCR) can recognize specific antigen, activate corresponding sensitized lymphocytes which make mature lymphocytes convert into lymphoblasts and grow from G_0_ stage into G_1_ stage with the synthesis of more protein, DNA, and RNA. So if ^3^H-TdR is added to the lymphocyte culture medium, it can be taken by the newly synthesized DNA, then the function of lymphocyte proliferation can be easily estimated by detection the amount of absorbed ^3^H-TdR.

CAUTION: *When working with human blood, cells, or infectious agents, biosafety practices must be followed.*

NOTE: *All solutions and equipment coming into contact with cells must be sterile, and proper sterile technique must be used accordingly.*

**B. Material requested:**

Heparinized blood

Ficoll-Hypaque solution (density1.075-1.092g/liter)(the Second Shanghai Chemical Reagent Corporation)

FCS

Completed RPMI 1640 medium

Scintillation fluid:

PPO 5 g

POPOP 0.5 g

Dimethylbenzene to 1000ml

^3^H-TdR( the Shanghai Nuclear Institute)

15ml conical centrifuge tubes

Cryotubes

Centrifuge with Sorvall H-1000 B rotor (or equivalent Temperature controlled centrifuge)

**C. Procedure:**

1. Take (3 mL) heparinized blood samples, centrifuge at 1500rpm for 15 min, collect blood serum and store frozen for serological assays.
2. Overlay the diluted blood sample gradually with Ficoll-hypaque (100% of blood volume) in 20 ml disposable centrifuge tubes.
3. Centrifuge the gradient at 800g for 20 minutes at room temperature to separate the peripheral mononuclear cells (PBMCs).
4. Collect after centrifugation the interface containing mononuclear cells with a Pasteur-pipette.
5. Transfer then PBMCs into a 10 ml disposable conical centrifuge tube.
6. Wash the cells two times with RPMI 1640 using 200g centrifugation for 10 minutes at 10°C.
7. Determine cell numbers for each sample by counting with an automatic cytometry (XE-2100 Sysmex).
8. Adjust the mononuclear cell concentration to 1×10^6^/ml with RPMI 1640 which contain 10% FCS, 10mmol/L Hepes, 1mmol/L sodium pyruvate, 2mmol/L glutamine, 0.075%NaHCO_3_, 100μg/mL streptomycin, and 100u/mL penicillin. Add 1×10^5^cells/100μL /well to the 96-well microtiter plate in triplicate and 100μL appropriate antigen PFCP-2/well, at the same time leave the negative control without adding antigen.
9. Place the microtiter plates in a humidified 37℃, 5%CO_2_ incubator for 60 hr, add 1μci ^3^H-TdR/50μL to each well.
10. Return the plates to CO_2_ incubator to pulse 12hr. Harvest cells using a semi-automated sample harvester and measure cpm in β scintillation counter.
11. Compute the data as the ratio of cpm of stimulated and control cultures. This is done by dividing the arithmetic mean of cpm from stimulated cultures by the arithmetic mean of cpm from control cultures. The results are refered to “ SI “( Stimulation Index).
12. The arithmetic mean of cpm from stimulated cultures－base line of the machine

SI=

The arithmetic mean of cpm from control cultures－base line of the machine

#

**APPENDIX 4:**

**重组疟疾疫苗I期临床研究**

**数据安全性监查小组评估意见表**

**[Assessment table for Phase I Clinical Trial of Malaria Vaccine from DSMB]**

| 接种日期 Vaccination date | □day 0  □day 60  □day 180 | 接种人数 Vaccination Number | □ 3 cases  □ 9 cases |
| --- | --- | --- | --- |
| 接种剂量 Vaccination dose | □20μg □50μg □100μg □200μg | | |
| 不良反应  （具体描述）  **Adverse Event**  **(description)** | 发生严重不良事件例数(Numbers of SAEs,see protocol 8.2.1)  □ 未发生(no) □1例 □2例 □3例  发生重度不良事件例数(Number of severe AEs)  □ 未发生 □1例 □2例 □3例  重度不良事件(grade 3)发生率  [Rate of severe AEs(grade 3),52 cases as demoninator]  □ ≤ 6% □ > 6% | | |
| 评估意见  **Assessment** | - 继续进行下一阶段临床研究   Go to the higher dose group. | | |
|  | - 研究方案需修改、调整后方可继续进行下一阶段临床研究   Protocol should be amended before going to the higher dose group. | | |
|  | - 终止临床研究   Trial should be stopped. | | |
| 签 字  **Signature** |  | | |
| 日 期 Date |  | | |

注：该评估表一经填写完毕，签字后立即传真至主要研究者胡晋红教授处，传真号码（0086）21 25070665或 临床观察员解飞，传真号码为（0086）21 25070673。

# Notice: Please FAX the table with signature of DSMB member immediately to PI-Prof. Jinhong Hu, whose phone number is （0086）21 25070665, or to clinical monitor-Fei Xie, whose phone number is (0086)21 58993709.
